# Supplementary material for: Developing and externally validating a machine learning risk prediction model for 30-day mortality after stroke using national stroke registers in the UK and Sweden
Source: BMJ Open. 2023 Nov 15;13(11):e069811. doi: 10.1136/bmjopen-2022-069811 (PMC10660948; doi:10.1136/bmjopen-2022-069811)
Supplement: Supplementary data [file bmjopen-2022-069811supp001.pdf]

Supplementary Material

Table A, Missing values in the variables and the imputation methods

	SSNAP		Riksstroke (in-hospital mortality)	
	**Missing (%)**	**Imputation method**	**Missing (%)**	**Imputation method**
Age groups	-	-	-	-
Male	-	-	-	-
Hypertension	-	-	0.5%	Multiple imputation
AF	-	-	0.2%	Multiple imputation
Diabetes	-	-	0.3%	Multiple imputation
Previous Stroke or TIA	-	-	0.3%	Multiple imputation
NIHSS at arrival	-	-	42.9%	Not used in the model
Level of consciousness	-	-	1.5%	Multiple imputation after splitting the non alert level to two levels
Pre-stroke mRS			5.0%	multiple imputation

anticoagulation prior to stroke if AF	58.9%	Unknown as a new category	-	-
Type of stroke	0.7%	Unknown as a new category	-	-

Abbreviation: AF: Atrial Fibrillation; TIA: transient ischaemic attack; NIHSS: National Institutes of Health Stroke Scale; mRS: modified Rankin Scale.

Table B: Brier score, AUC with 95% Confidence Interval (CI) for SSNAP development set and internal validation set.

	Brier score (95% CI)		AUC (95% CI)	
	SSNAP development set	SSNAP validation set	SSNAP development set	SSNAP validation set
LR	0.081 (0.080, 0.082)	0.079 (0.78, 0.81)	0.843 (0.841, 0.845)	0.845 (0.841, 0.849)
LR with elastic net and interaction terms	0.080 (0.079, 0.081)	0.078 (0.77, 0.80)	0.845 (0.843, 0.847)	0.848 (0.844, 0.851)
XGBoost	0.079 (0.079, 0.080)	0.078 (0.77, 0.79)	0.848 (0.846, 0.850)	0.849 (0.845, 0.853)

Table C, Brier score, AUC with 95% Confidence Interval (CI) for 2019 temporal validation and RiksStroke external validation with in-hospital mortality and all mortality.

	Brier score (95% CI)			AUC (95% CI)		
	SSNAP	RiksStroke (in-hospital mortality)	RiksStroke (all mortality)	SSNAP	RiksStroke (in-hospital mortality)	RiksStroke (all mortality)
LR	0.078 (0.076, 0.080)	0.066 (0.065, 0.067)	0.076 (0.075, 0.077)	0.847 (0.841, 0.853)	0.861 (0.857, 0.864)	0.859 (0.856, 0.862)
LR with elastic net and interaction terms	0.078 (0.076, 0.080)	0.067 (0.066, 0.068)	0.077 (0.076, 0.078)	0.850 (0.844, 0.856)	0.862 (0.859, 0.866)	0.861 (0.858, 0.864)
XGBoost	0.077 (0.075 ,0.079)	0.066 (0.065, 0.067)	0.077 (0.076, 0.078)	0.852 (0.846, 0.858)	0.861 (0.858, 0.865)	0.860 (0.857, 0.863)

Table D, the coefficients of variables from logistic regression

Variables	Coefficients
**loss of consciousness**	**1.26**

**type of stroke Haemorrhage**	**0.92**
**Inpatient at time of stroke**	**0.47**
**atrial fibrillation**	**0.46**
**type of stroke Unknown**	**0.32**
**Age (Groups by 5)**	**0.22**
**hour of admission 04 to 08**	**0.17**
**previous stroke/TIA**	**-0.16**
**prestroke mRS**	**0.16**
**hour of admission 12 to 16**	**-0.14**
**hypertension**	**-0.12**
**Male**	**0.11**
**admission Friday**	**-0.10**
**diabetes**	**0.10**
**hour of admission 16 to 20**	**-0.09**
**admission Monday**	**-0.08**
**prior anticoagulation if AF Nobut**	**0.07**
**admission Tuesday**	**-0.05**
**admission Wednesday**	**-0.05**

**admission Thursday**	**-0.05**
**admission Saturday**	**-0.03**
**hour of admission 08 to 12**	**-0.03**
**prior anticoagulation if AF Yes**	**-0.02**
**hour of admission 20 to 24**	**0.02**
**prior anticoagulation if AF Unknown**	**-0.005**

Abbreviation: AF: Atrial Fibrillation; TIA: transient ischaemic attack; mRS: modified Rankin Scale.

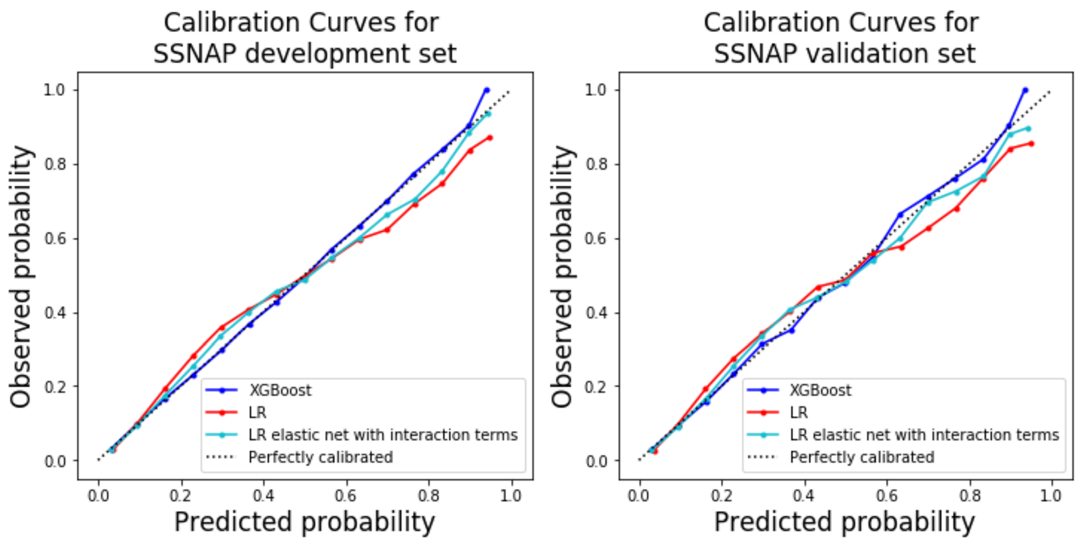

Figure A: Calibration curves for all models on development set and validation set of SSNAP cohort.

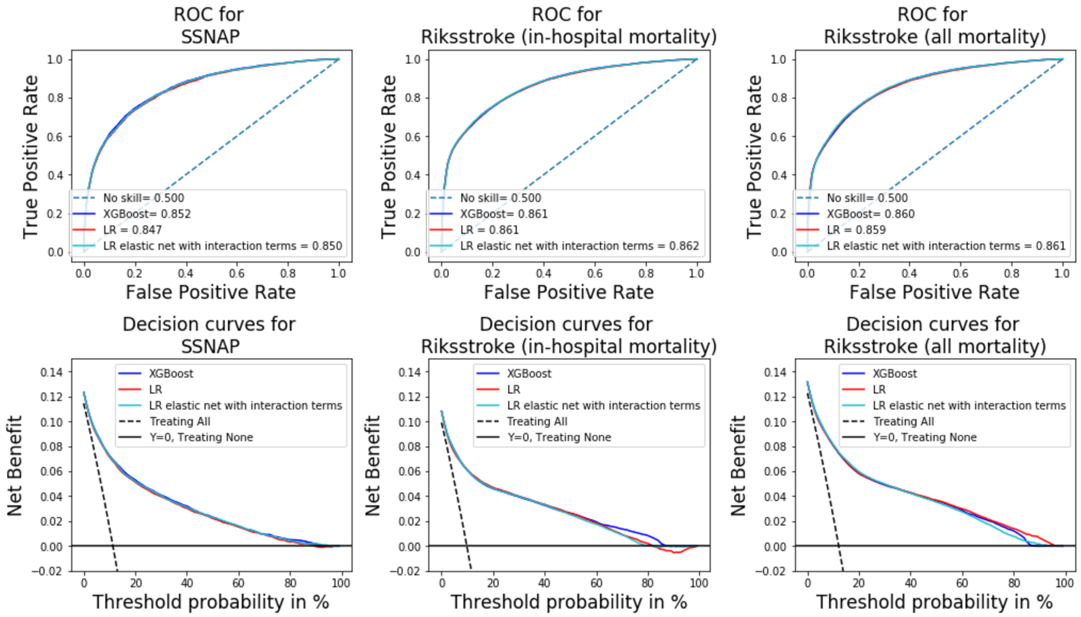

Figure B: ROC and decision curves for all models on 2019 temporal validation of SSNAP cohort (left) and external validation using Riksstroke with in-hospital mortality (middle) and all mortality (right)

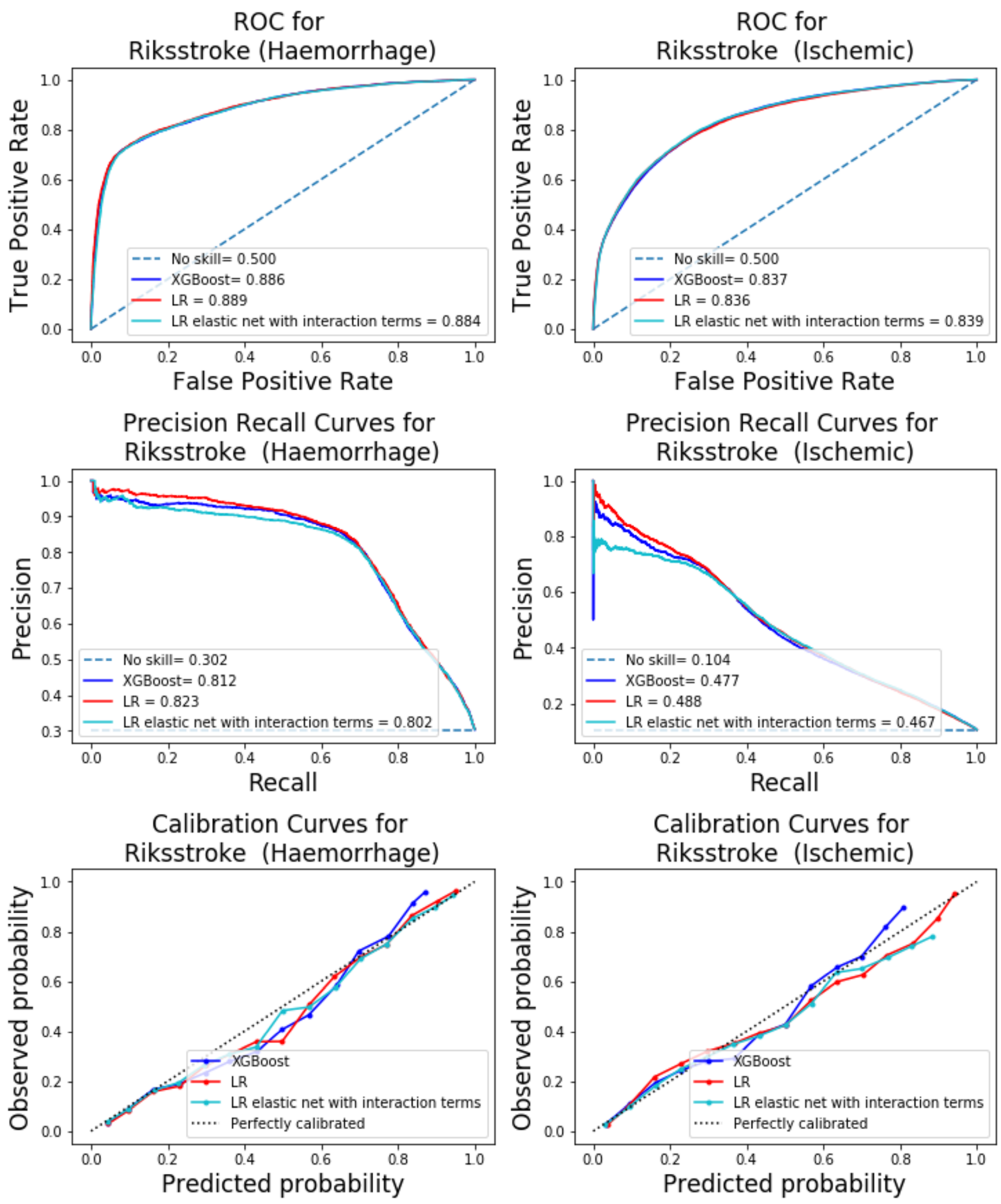

Figure C: AUC-ROC, PR-curve, and Calibration-curve for all mortalities (Haemorrhage (left) vs Ischemic (right) patients) in Riksstroke

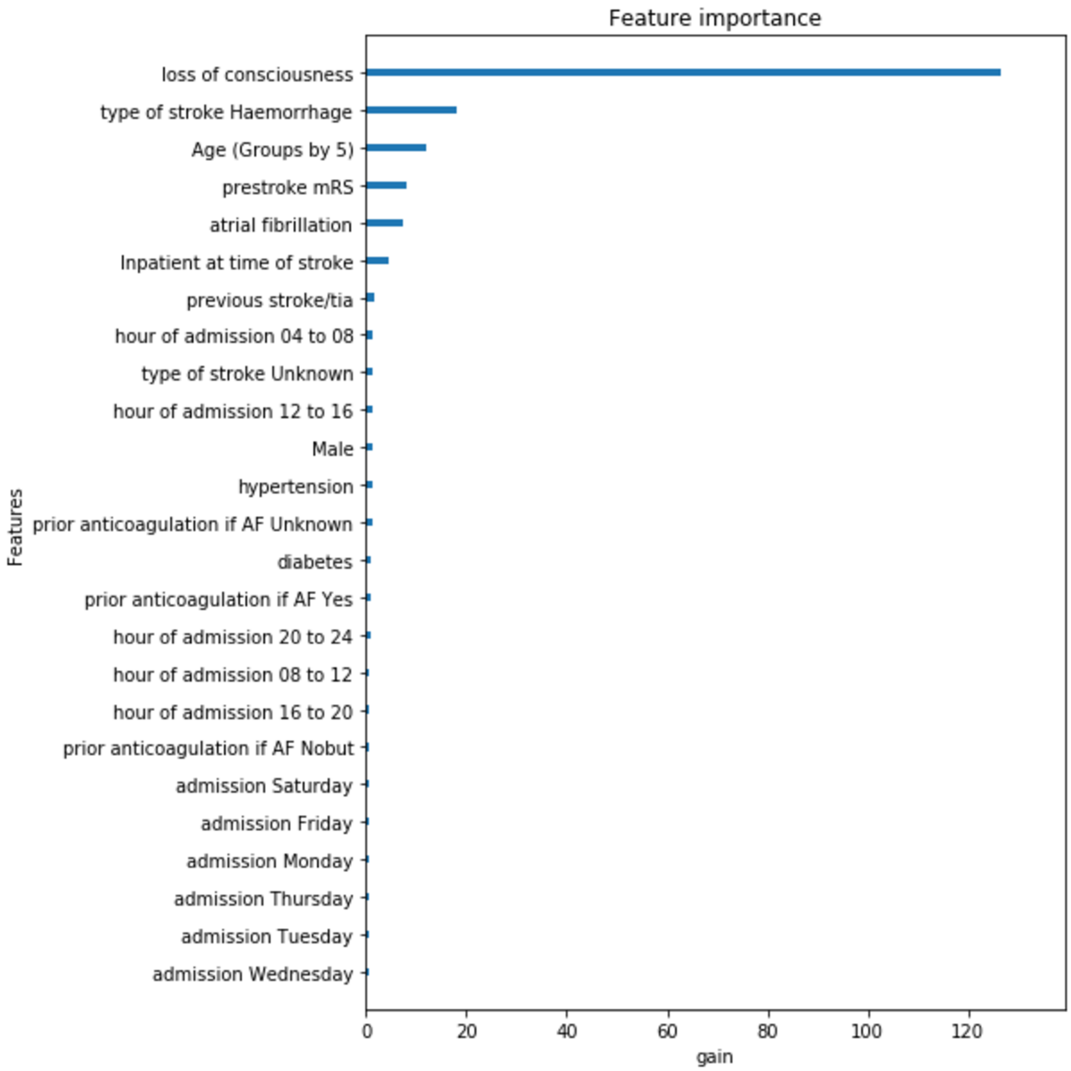

Figure D: Feature importance from XGBoost using gain as measurement of feature importance
