# Supplementary material for: Developing and externally validating a machine learning risk prediction model for 30-day mortality after stroke using national stroke registers in the UK and Sweden
Source: BMJ Open. 2023 Nov 15;13(11):e069811. doi: 10.1136/bmjopen-2022-069811 (PMC10660948; doi:10.1136/bmjopen-2022-069811)
Supplement: Author's manuscript [file bmjopen-2022-069811.draft_revisions.pdf]

BMJ Open

BMJ Open is committed to open peer review. As part of this commitment we make the peer review history of every article we publish publicly available.

When an article is published we post the peer reviewers' comments and the authors' responses online. We also post the versions of the paper that were used during peer review. These are the versions that the peer review comments apply to.

The versions of the paper that follow are the versions that were submitted during the peer review process. They are not the versions of record or the final published versions. They should not be cited or distributed as the published version of this manuscript.

BMJ Open is an open access journal and the full, final, typeset and author-corrected version of record of the manuscript is available on our site with no access controls, subscription charges or pay-per-view fees (http://bmjopen.bmj.com).

If you have any questions on BMJ Open's open peer review process please email info.bmjopen@bmj.com

BMJ Open

Developing and externally validating a machine learning risk prediction model for 30-day mortality after stroke using national stroke registers in the UK and Sweden

Journal:	*BMJ Open*
Manuscript ID	bmjopen-2022-069811
Article Type:	Original research
Date Submitted by the Author:	10-Nov-2022

Complete List of Authors:	Wang , Wenjuan; King's College London<br>Otieno, Josline; Umeå University, Department of Statistics<br>Eriksson, Marie; Umea Universitet, Statistics<br>Wolfe, Charles; King's College London<br>Curcin, Vasa; King's College London<br>Bray, Benjamin; King's College London

Keywords:	Stroke < NEUROLOGY, Quality in health care < HEALTH SERVICES ADMINISTRATION & MANAGEMENT, NEUROLOGY, STATISTICS & RESEARCH METHODS

SCHOLARONE™
Manuscripts

I, the Submitting Author has the right to grant and does grant on behalf of all authors of the Work (as defined in the below author licence), an exclusive licence and/or a non-exclusive licence for contributions from authors who are: i) UK Crown employees; ii) where BMJ has agreed a CC-BY licence shall apply, and/or iii) in accordance with the terms applicable for US Federal Government officers or employees acting as part of their official duties; on a worldwide, perpetual, irrevocable, royalty-free basis to BMJ Publishing Group Ltd ("BMJ") its licensees and where the relevant Journal is co-owned by BMJ to the co-owners of the Journal, to publish the Work in this journal and any other BMJ products and to exploit all rights, as set out in our licence.

The Submitting Author accepts and understands that any supply made under these terms is made by BMJ to the Submitting Author unless you are acting as an employee on behalf of your employer or a postgraduate student of an affiliated institution which is paying any applicable article publishing charge ("APC") for Open Access articles. Where the Submitting Author wishes to make the Work available on an Open Access basis (and intends to pay the relevant APC), the terms of reuse of such Open Access shall be governed by a Creative Commons licence – details of these licences and which Creative Commons licence will apply to this Work are set out in our licence referred to above.

Other than as permitted in any relevant BMJ Author's Self Archiving Policies, I confirm this Work has not been accepted for publication elsewhere, is not being considered for publication elsewhere and does not duplicate material already published. I confirm all authors consent to publication of this Work and authorise the granting of this licence.

For peer review only

Developing and externally validating a machine learning risk prediction model for 30-day mortality after stroke using national stroke registers in the UK and Sweden

Wenjuan Wang[1], Josline Otieno[2], Marie Eriksson[2], Charles D Wolfe[1,3], Vasa Curcin[1,3], Benjamin Bray[1]

1 School of Population Health and Environmental Sciences, King's College London, London, UK

2 Department of Statistics, Umeå School of Business, Economics and Statistics (USBE), Umeå University, Umeå, Sweden

3 NIHR Biomedical Research Centre, Guy's and St Thomas' NHS Foundation Trust

Corresponding author: Dr Wenjuan Wang, Addison House 3.07, Guy’s Campus, London SE1 1UL, UK. Email: wenjuan.wang@kcl.ac.uk

Keywords: stroke, mortality, risk prediction, quality improvement, external validation

Word count: 4558

For peer review only

Abstract

Objectives

We aimed to develop and externally validate a generalisable risk prediction model for 30-day stroke mortality suitable for supporting quality improvement analytics in stroke care using large nationwide stroke registers in the United Kingdom and Sweden.

Methods

Data from the Sentinel Stroke National Audit Program (SSNAP) in England, Wales, and Northern Ireland (2013-2019) were used for developing and temporally validating the model, and data from the national Swedish stroke register (Riksstroke 2015-2020) were used for external validation. Models were developed with the variables available in both registries and using logistic regression (LR), LR with elastic net and interaction terms, and XGBoost. Performances were evaluated with discrimination, calibration, and decision curves.

Results

In total, 488,497 stroke patients with 12.4% 30-day in-hospital mortality were used for developing and temporally validating the model in the UK. A total of 128,360 stroke patients with 10.8% 30-day in-hospital mortality and 13.1% all mortality were used for external validation in Sweden. In the SSNAP temporal validation set, the final XGBoost model achieved the highest area under the ROC curve (AUC) (0.852 (95% CI: 0.848-0.855)) and was well-calibrated. The performances on the external validation in Riksstroke were as good and achieving AUC at 0.861 (95% CI: 0.858, 0.865) for in-hospital mortality. For Riksstroke, the models slightly overestimated the risk for in-hospital mortality, whilst they were better calibrated at the risk for all mortality.

Conclusion

The risk prediction model was accurate and externally validated using high quality registry data which is potentially suitable to be deployed as part of quality improvement analytics in stroke care to enable the fair comparison of stroke mortality outcomes across hospitals and health systems across countries.

For peer review only - http://bmjopen.bmj.com/site/about/guidelines.xhtml

Strengths and limitations of this study

This is the first cross-national study to develop and externally validate a machine learning based risk prediction model for 30-day mortality after stroke suitable for use in care quality registers.

The models were built and externally validated with very large and comprehensive stroke datasets and both of the data sources include a nearly complete population of hospitalised stroke in England and Wales (SSNAP) and Sweden (Riks-Stroke) reducing the risk of bias.

The models were built with robust approaches and reported according to the TRIPOD reporting guidelines with several terms adjusted for ML studies such as hyperparameter turning and final models were published and have been made freely available for download for future external validation and use.

The main limitation are 1) death outcomes were limited to inpatient mortality and was not possible to ascertain deaths occurring outside hospital within 30 days for SSNAP data, 2) NIHSS as a main risk predictor for mortality was not available in Riksstroke, and 3) the variables were limited to the ones that are available in both SSNAP and Riksstroke.

For peer review only

Introduction

Risk prediction models are valuable tools in benchmarking health care outcomes across different hospitals or countries, thereby contributing to improved clinical practice[1]. These models are required to adjust for differences in the case mix of patients to allow for an unbiased comparison of stroke outcomes, and can be used to identify patients at high risk of poor outcomes. Benchmarking and comparing stroke outcomes across hospitals and health systems is widely used as part of quality improvement initiatives and quality registers in stroke[2], but depends on accurate adjustment of case mix differences to be valid.

Prediction models using machine learning (ML) algorithms are now being widely used in healthcare (e.g. in the interpretation of medical imaging data) but the potential of ML to improve the accuracy of analytics used for measuring and comparing the quality of stroke care services is largely unexplored. Moreover, a systematic review on the application of ML methods in predicting outcomes of stroke based on structured data found that few studies met basic reporting standards for clinical prediction tools[3]. Major issues included lack of external validation, and the non-availability of the final model for external validation[3]. External validation (i.e. evaluating the accuracy of the predictions in data from a different source that used to develop the model) is especially important in assessing the generalizability of the model before it is used in other settings.

Traditionally, logistic regression (LR) has been used for risk predictions of stroke outcome. It has been suggested that ML might outperform clinical prediction models based on LR because they make fewer assumptions and can learn complex relationships between predictors and outcomes[4]. However, there are also studies showing that no evidence of superior performance of ML over LR and improvements in methodology and reporting are needed for studies that compare modelling algorithms[5]. In a previous study, we compared updated LR models from Bray et al. 2014[6] and a ML algorithm (XGBoost) for predicting 30-day mortality after stroke and found that XGBoost performed the best[7]. Existing scores for 30-day mortality prediction, e.g., PLAN[8] and IScore[9] were not compared in the previous study due to lack of certain variables.

Models developed and validated in one nation might not perform well with another nation due to the differences in the population and health systems. In this study, we aimed to develop and externally validate a generalisable risk prediction model for 30-day mortality after stroke using large nationwide stroke registers in the United Kingdom and Sweden. The intention was to

For peer review only - http://bmjopen.bmj.com/site/about/guidelines.xhtml

develop a model developed and externally validated in high quality registry data which could be made publicly available to support stroke research (e.g. cross national or multi-centre comparisons of stroke mortality) or be deployed as part of quality improvement analytics initiatives in stroke.

Methods

This study is reported according to the TRIPOD guidelines: transparent reporting of a multivariable prediction model for individual prognosis or diagnosis[10].

Data source

Data from SSNAP for model development and internal validation

Data for developing the models were from Sentinel Stroke National Audit Programme (SSNAP), the national registry of stroke care in England, Wales and Northern Ireland. SSNAP is a Healthcare Quality Improvement Partnership (HQIP) register for stroke care quality improvement. Data were collected prospectively, validated by clinical teams and entered into the SSNAP database via a secure web interface. SSNAP includes an estimated 95% of all adults admitted to hospital with acute stroke (ischaemic or primary intracerebral haemorrhage) in England, Wales and Northern Ireland. The original dataset from SSNAP was collected from 333 teams across England, Wales and Northern Ireland between April 1, 2013 to June 30, 2019. For the generalisation of the model to the population, all patients were included and no specific exclusion criteria for stroke patients.

Data from Riksstroke for external validation

We then used data from the Swedish stroke register (Riksstroke) for external validation of the developed models. Riksstroke is a national quality register, monitoring stroke care and outcome in adult patients. Details about the register and included variables have been published previously[11] and the forms can be found on Riksstroke's webpage (https://www.riksstroke.org/forms/).

In short, all hospitals admitting acute stroke (ischaemic, primary intracerebral haemorrhage, or unspecified type of stroke) patients in Sweden participate (currently 72 hospitals). Riksstroke is estimated to include more than 90% of all acute stroke patients treated in hospital. The register includes information from the entire chain of stroke care, including primary prevention,

For peer review only - http://bmjopen.bmj.com/site/about/guidelines.xhtml

acute management, rehabilitation, secondary prevention, and family and community support. The dataset for external validation included patients who experienced stroke between January 1, 2015 to December 31, 2020**.**

Availability of data and materials

The data that support the findings of this study are available from SSNAP (www.strokeaudit.org) and Riksstroke (www.riksstroke.org) but restrictions apply to the availability of these data, which were used under license for the current study, and so are not publicly available. Data are however available from the SSNAP and Riksstroke upon reasonable request and with permission of HQIP and Riksstroke, respectively.

Study outcome

For models developed with data from the SSNAP registry, the outcome was all-cause 30-day in-hospital mortality post-stroke. All patients had in-hospital status due to the collection procedure in SSNAP. Out-hospital deaths were not available for analysis.

For the external validation with data from Riksstroke, date of death was retrieved for all patients by individual linkage with the national Cause of Death register, managed by the National Board of Health and Welfare, using personal identification numbers. Hence, all 30-day mortality included both in- and out-of-hospital deaths.

The developed models with SSNAP data were externally validated with both Riksstroke in-hospital deaths and all deaths as outcomes. Performances for in-hospital death were compared between SSNAP and Riksstroke. Comparisons between all deaths and in-hospital death in Riksstroke were conducted as a sensitivity analysis.

Predictor variables

In total, 65 variables were available from SSNAP. Details on how each risk predictor was measured have been presented in a previous study for variables in SSNAP[7] and a previous study for variables in Riksstroke[11]. Subsequently, 13 variables were used to build and validate prediction models of 30-day mortality. The variable selection was done based on the clinical importance, literature review, and availability in both the SSNAP and Riksstroke registries. Different clinical covariates for prediction included age (band by 5), sex, inpatient at time of stroke, hour of admission, day of week of admission, comorbities such as hypertension, AF, diabetes, previous stroke or transient ischaemic attack (TIA), and anticoagulation prior to

For peer review only - http://bmjopen.bmj.com/site/about/guidelines.xhtml

stroke if AF, pre-stroke modified Rankin Scale (mRS), loss of consciousness, and type of stroke (ischemic or primary intracerebral haemorrhage). The National Institutes of Health Stroke Scale (NIHSS) was not used as a variable/predictor due to the high missingness in Riksstroke (42.9%). Bray et al. 2014 showed that the level of consciousness component of the NIHSS provides a good approximation of the full NIHSS for mortality prediction[2].

Two predictors were recorded differently in SSNAP and Riksstroke, i.e. level of consciousness and pre-stroke mRS. In SSNAP, level of consciousness was assessed based on the first item of the NIHSS which has four levels, i.e., 0 = alert, 1 = not alert but arousable by minor stimulation, 2 = not alert and requires repeated stimulation to attend, and 3 = unconscious. In Riksstroke, level of consciousness was recorded in three categories where alert corresponds to level of consciousness 0 in SSNAP, not alert to level of consciousness 1-2 in SSNAP, and unconscious level of consciousness 3 in SSNAP. To obtain 4 categories in Riksstroke, a total NIHSS score ≤13 vs. >13 was used to split the mid category (not alert). The cutoff was chosen based on the NIHSS score distribution in Riksstroke and previous studies showing good discrimination properties for 30-day stroke mortality[12]. In Riksstroke pre-stroke mRS was estimated using a previously developed algorithm[13] which has four categories with the first category corresponding to 0-2 and the next three categories corresponding to 3, 4, 5 in the pre-Stroke mRS in SSNAP. The first category was imputed to be 0 in Riksstroke for the external validation.

Missing data

Missing data were handled using different methods according to assumptions of missing mechanism after consulting the SSNAP team and clinicians. Variables with more than 80% missingness were discarded. For categorical variables with missing by design/not applicable assumption, missing values were added as a new category. Missing indicator was used for missing by design/not applicable continuous variables. After these, Multiple Imputation with Chained Equations (MICE)[14] was used to impute variables with missing at random assumption for the development dataset. All available variables except for the discarded ones were used in MICE. Five datasets were imputed using MICE and aggregated into one using the median for SSNAP with all the variables available in the dataset and Riksstroke dataset. Details for handling missing data in SSNAP were presented in a previous study[7].

Model building and evaluation

For peer review only - http://bmjopen.bmj.com/site/about/guidelines.xhtml

After variable selection and imputation of missing values, the variables were pre-processed. Details of preprocessing and coding of variables were presented in a previous study[6] and in the GitHub repository (https://github.com/WenjuanW/Risk_Prediction_of_Post-Stroke_30-day_Mortality_SSNAP_and_RiksStroke). Descriptive statistics were used to compare the characteristic of death/alive at 30-day across the entire datasets and between SSNAP and Riksstroke.

Models were developed/trained with data from SSNAP using 80% randomly selected patients from 2013 to 2018, validated on the remaining 20% data from 2013 to 2018, and temporally validated on data from 2019. Subsequently, models were externally validated using data from Riksstroke from between January 1, 2015 to December 31, 2020.

Due to the lack of final models from other ML studies and lack of certain variables for risk scores, we could not externally validate existing ML models and risk scores. From the comparisons of existing Logistic regression (LR) models with ML studies, XGBoost performed the best[7]. Thus, new models were built with LR, LR with elastic net[15] with interaction terms, and eXtreme Gradient Boosting (XGBoost)[16] using the mutual risk predictors that were available in both countries in order to externally validate the models. Best hyperparameters (a parameter that is predefined by the user to control the learning process) were selected on the development set with grid search or random search and cross-validation (CV). Detailed hyperparameter tuning strategy was presented in a repository on Github that was built for this study (https://github.com/WenjuanW/Risk_Prediction_of_Post-Stroke_30-day_Mortality_SSNAP_and_RiksStroke).

Brier score[17] was used as an overall summative measure of predictive performance. Discrimination was measured by area under the ROC curve (AUC). Calibration was visually assessed with calibration plots[15] rather than Hosmer-Lemeshow test which has a number of drawbacks, including limited power, poor interpretability and is not recommended[19]. Python functions for calculating these measurements were presented in the Github repository.

Comparisons of AUCs and Brier scores within each set were conducted with Delong's test and paired t-test respectively. To compare AUCs between the performances on SSNAP and Riksstroke, an independent group t-test was used for both AUCs and Brier scores with 500 bootstraps for each measurement.

Due to the low outcome rate, the dataset is imbalanced at predicting the outcomes. In this case, AUC cannot fully reflect the performances of the models on positive predictive rate (precision)

For peer review only - http://bmjopen.bmj.com/site/about/guidelines.xhtml

and true positive rate (recall). Therefore, precision and recall were assessed via area under the precision-recall curves and the precision-recall curves.

Clinical utility was assessed with decision curve analysis[20] which graphically shows the net benefit obtained by applying the strategy of treating an individual if and only if predicted risk is larger than a threshold in function of the threshold probability. Threshold equals to 0 means treating all since all predicted risk will be larger than 0. Threshold equals to 1 means treating none since all predicted risk will be smaller than 1. All analyses were conducted using Python 3.7.4.

As stroke type is a critical factor for outcomes of stroke patients, we subsequently performed a subgroup analysis to investigate the performance of the developed models for patients with different stroke types i.e. infarction and primary intracerebral haemorrhage.

Sensitivity analysis

External validation on all 30 day mortality (i.e including deaths outside of hospital) in Riksstroke was conducted as a sensitivity analysis to check the impact of using only in-hospital mortality for developing the models. Another sensitivity analysis was done using mRS = 1 as the level representing 0-2 in previous stroke mRS variable in Riksstroke.

Results

Participants

The characteristics of 488,947 patients from SSNAP and 128,360 patients from Riksstroke were reported in Table 1. In total, 60,362 (12.4%) patients from SSNAP and 13,873 (10.8%) from Riksstroke died within 30 days in hospital and (13.1% for all mortality within 30 days). In both SSNAP and Riksstroke, patients who died within 30 days were older, and were more likely to have cardiovascular risk factors, impaired functional status prior to stroke, haemorrhagic stroke, or being unconscious upon arrival to hospital (Table 1).

The patient case-mix in SSNAP and Riksstroke was similar with respect to age distribution. Riksstroke had a high percentage of males for patients who died at 30 days. A higher frequency in Riksstroke compared to SSNAP was observed in hypertension (63.0% vs 54.2%), AF (28.9% vs 19.7%), diabetes (22.4% bs 20.9%), previous stroke or TIA (27.4% vs 26.5%), and haemorrhage (13.1% vs 11.5%). The difference in these risk factors is even larger for the patients who died with 30-days after stroke with hypertension (67.4% vs 53.5%), AF (43.5%

For peer review only - http://bmjopen.bmj.com/site/about/guidelines.xhtml

vs 33.0%), diabetes (22.6% bs 20.2%), previous stroke or TIA (31.6% vs 28.3%), and haemorrhagic stroke (33.8% vs 27.2%). A lower percentage of high level of consciousness (level 3) was observed in Riksstroke compared to SSNAP (4.6% vs 6.8%). On the contrary, a higher percentage of high levels of previous stroke mRS was found in Riksstroke compared to SSNAP (2.82% vs 1.8% for level 5, 9.2% vs 6.2% for level 4, and 14.8% vs 11.7% for level 3).

Summary of missing percentages and imputation methods for all predictors were presented in Supplementary Table A. In SSNAP, only type of stroke predictor had missing values of 0.7% and the missing values were taken as a new category. In Riksstroke, pre-stroke mRS had 5.0% missing values and level of consciousness had 1.5% missing values. Missing percentages for comorbidity variables were small (less than 0.5%).

Table 1, Patients characteristics in SSNAP and Riksstroke

	SSNAP			Riksstroke (in-hospital mortality)		
	**Overall**	**Alive at 30 days**	**Dead at 30 days**	**Overall**	**Alive at 30 days**	**Dead at 30 days**
No. cases (%)	488,947	428,585 (87.6)	60,362 (12.4)	128,360	114,487 (89.2)	13,873 (10.8)
Age groups (%)						
15-60	76,941 (15.7)	74,238 (17.3)	2,703 (4.5)	15,586 (12.1)	14,958 (13.1)	628 (4.5)
61-70	84,484 (17.3)	79,109 (18.5)	5,375 (8.9)	23,186 (18.1)	21,782 (19.0)	1,404 (10.1)
71-80	136,728 (28.0)	122,393 (28.6)	14,335 (23.7)	41,396 (32.3)	37,689 (32.9)	3,707 (26.7)
81+	190,794 (39.0)	152,845 (35.7)	37,949 (62.9)	48,192 (37.5)	40,058 (35.0)	8,134 (58.6)
Male (%)	249,291	223,849	25,442 (42.1)	68,985	62,428	6,557

For peer review only - http://bmjopen.bmj.com/site/about/guidelines.xhtml

	SSNAP			Riksstroke (in-hospital mortality)		
	(51.0)	(52.2)		(53.7)	(54.5)	(47.3)
Hypertension (%)	264,806 (54.2)	232,505 (54.2)	32,301 (53.5)	80,824 (63.0)	71,481 (62.4)	9,343 (67.4)
AF (%)	96,354 (19.7)	76,417 (17.8)	19,937 (33.0)	37,090 (28.9)	31,057 (27.1)	6,033 (43.5)
Diabetes (%)	102,324 (20.9)	90,104 (21.0)	12,220 (20.2)	28,722 (22.4)	25,589 (22.4)	3,133 (22.6)
Previous Stroke or TIA (%)	129,462 (26.5)	112,358 (26.2)	17,104 (28.3)	35,145 (27.4)	30,757 (26.9)	4,388 (31.6)
NIHSS at arrival (median [IQR])	4.00 [2.0, 10.0]	4.00 [2.0, 8.0]	17.00 [7.0, 23.0]	3.00 [1.0, 8.0]	3.00 [1.0, 7.0]	16.00 [9.0, 23.0]
Level of consciousness (%)*						
0 (alert)	391,065 (80.0)	379,085 (88.5)	11,980 (19.8)	106,474 (83.0)	101,706 (88.8)	4,768 (34.4)
1 (not alert)	419,16 (8.6)	32,715 (7.6)	9,201 (15.2)	14,067 (11.0)	9,459 (8.3)	4,608 (33.2)
2 (not alert)	22,635 (4.6)	11,790 (2.8)	10,845 (18.0)			
3 (unconscious)	33,331 (6.8)	4,995 (1.2)	28,336 (46.9)	5,930 (4.6)	1,759 (1.5)	4,171 (30.1)
Pre-stroke mRS (%)						

For peer review only

	SSNAP			Riksstroke (in-hospital mortality)		
0	266,409 (54.5)	245,979 (57.4)	20,430 (33.8)			
1	75,661 (15.5)	66,940 (15.6)	8,721 (14.4)	87,593 (68.2)	81,871 (71.5)	5,722 (41.3)
2	50,795 (10.4)	42,592 (9.9)	8,203 (13.6)			
3	56,994 (11.7)	44,601 (10.4)	12,393 (20.5)	18,942 (14.8)	16,563 (14.5)	2,379 (17.2)
4	30,276 (6.2)	22,339 (5.2)	7,937 (13.1)	11,763 (9.2)	9,483 (8.3)	2,280 (16.4)
5	8,812 (1.8)	6,134 (1.4)	2,678 (4.4)	3,623 (2.8)	2,720 (2.4)	903 (6.5)
Haemorrhage (%)	55,758 (11.5)	39,472 (9.3)	16,286 (27.2)	16,757 (13.1)	12,074 (10.6)	4,683 (33.8)

Abbreviation: AF: Atrial Fibrillation; TIA: transient ischaemic attack; NIHSS: National Institutes of Health Stroke Scale; mRS: modified Rankin Scale. * Level of consciousness is measured differently between SSNAP. In SSNAP, Level of consciousness has four levels (0 = alert, 1 = not alert but arousable by minor stimulation, 2 = not alert and requires repeated stimulation to attend, and 3 = unconscious). In Riksstroke, Level of consciousness has three levels (0 = alert, 1,2 = not alert, 3 = unconscious).

Table 2, Brier score, AUC with 95% Confidence Interval (CI) for 2019 temporal validation in SSNAP and Riksstroke external validation with in-hospital mortality.

	Brier score (95% CI)		AUC (95% CI)	
	SSNAP	Riksstroke	SSNAP	Riksstroke
LR	0.078 (0.076, 0.080)	0.066 (0.065, 0.067)	0.847 (0.841, 0.853)	0.861 (0.857, 0.864)

For peer review only

LR with elastic net and interaction terms	0.078 (0.076, 0.080)	0.067 (0.066, 0.068)	0.850 (0.844, 0.856)	0.862 (0.859, 0.866)
XGBoost	0.077 (0.075 ,0.079)	0.066 (0.065, 0.067)	0.852 (0.846, 0.858)	0.861 (0.858, 0.865)

Model specification and performance

General characteristics for the development, validation and temporal validation set on all candidate variables are presented in previous study[7]. Specifications of the trained models and explanations on how to use them can be found in the repository on Github (https://github.com/WenjuanW/Risk_Prediction_of_Post-Stroke_30-day_Mortality_SSNAP_and_RiksStroke). All models performed similarly on training set and validation set indicating the models were not overfitting (Supplementary Table B) and calibrated well (Supplementary Figure A).

For the performances on 2019 temporal validation with SSNAP data (Table 2), XGBoost obtained the lowest Brier score of 0.077 (95% CI: 0.075 to 0.079) and the highest AUC of 0.852 (95% CI: 0.846 to 0.858) compared to LR with elastic net and interaction terms (Brier score: 0.078 (0.076, 0.080) and AUC: 0.850 (0.844, 0.856)) and LR (Brier score: 0.078 (0.076, 0.080) and AUC: 0.847 (0.841, 0.853)). The differences between the models were all significant (p-value < 0.001) even though small.

For the performances on external validation with Riksstroke data (Table 2) with in-hospital mortality, XGBoost achieved the lowest Brier score (0.066 (95% CI: 0.065 to 0.067) vs 0.067 (95% CI: 0.066 to 0.068) from LR with elastic net and interaction terms vs 0.066 (95% CI: 0.065 to 0.067) from LR, p-value < 0.001). In terms of AUC, XGboost achieved the slightly better performance compared to LR with elastic net and interaction terms (0.861 (95% CI: 0.858 to 0.865) vs 0.862 (95% CI: 0.859 to 0.866), p-value = 0.008) whilst better than LR (0.861 (95% CI: 0.857 to 0.864), p-value < 0.001). The performances of the models on Riksstroke data were slightly better than on SSNAP temporal validation data. The AUC between in-hospital and all-mortality in RiksStroke were similar (Supplementary Table C and Figure B)

From the precision-recall curves (Figure 1), the models were better at predicting precision on the Riksstroke data compared to on SSNAP data. Calibration plots in Figure 1 shows good

For peer review only - http://bmjopen.bmj.com/site/about/guidelines.xhtml

calibration for the 2019 temporal validation data in SSNAP with slightly underestimation of the risks for patients with medium risks (0.2-0.6). The models overestimated the risks for in-hospital mortality in RiksStroke for about 0.1 for medium risks patients whilst the overestimation was smaller for the all mortality predictions in RiksStroke. Decision curves on SSNAP 2019 temporal validation set and Riksstroke external validation set showed all models gained similar net benefits[20] (Supplementary Figure B).

For different stroke types, all models performed better on haemorrhagic patients than infarction patients on both SSNAP data (Figure 2) and RiksStroke data (Figure 3 for in-hospital mortality and Supplementary Figure C for all-mortality) in terms of AUC and area under precision-recall curves. Calibrations were similar between different stroke types (Figure 2, 3 and Supplementary Figure C).

According to the feature importance calculated from the XGBoost model (Supplementary Figure D), level of consciousness, type of stroke, age, prestroke mRS, and AF were the most important features in making the predictions which were in line with the literature. The coefficients from LR model (Supplementary Table D) also confirmed the importance of these variables.

Through the sensitivity analysis of using pre-stroke mRS=1 as the level representing 0-2 in previous stroke mRS variable in Riksstroke, we found no impact on the performances.

Figure 1: Precision-Recall curves (above) and Calibration curves (below) for all models on temporal validation of SSNAP cohort (left) and external validation using Riksstroke with in-hospital mortality (middle) and Riksstroke with all mortality (right)

Figure 2: AUC-ROC, PR-curve, and Calibration-curve for in-hospital mortalities (Haemorrhage (left) vs Ischemic (right) patients) in SSNAP temporal validation set

Figure 3: AUC-ROC, PR-curve, and Calibration-curve for in-hospital mortalities (Haemorrhage (left) vs Ischemic (right) patients) in Riksstroke

For peer review only - http://bmjopen.bmj.com/site/about/guidelines.xhtml

Discussion

This is the first cross-national study to develop and externally validate a ML-based risk prediction model for 30-day mortality after stroke suitable for use in care quality registers. Mortality is an important outcome after stroke and widely reported by stroke quality initiatives as a measure of quality and safety of acute stroke care, including in the Riks-Stroke and SSNAP national quality registers used in this study. The results of the internal validation in the UK and the external validation in Sweden were similar and together provide evidence that the model was accurate and generalisable across countries and data sources. This is promising for developing a single model that can be used to benchmark different hospitals/organisations in different countries and make fairer comparisons of stroke outcomes across countries.

Externally validated prediction models can be valuable tools in benchmarking health care outcomes across different hospitals[1] or countries and thereby contributing to improved clinical practice. This can be achieved by comparing the standardised mortality ratio (SMR)[20] which is calculated using the observed mortality rate and the predicted risk of mortality. A higher than expected SMR may be an indicator of poorer quality care, and there is strong evidence that higher quality acute stroke care (such as stroke unit based care[21]) reduces the risk of dying after stroke. The validity of this approach is however dependent on being able to make an accurate prediction of mortality, and hence it is important that the models used to generate these predictions are as accurate as possible and have been thoroughly validated.

We found that the performance of the model in external validation with Riksstroke data was in fact slightly better than in the temporal validation using data from SSNAP. This may be because the models performed more accurately in patients with haemorrhagic stroke and Riksstroke patients had a higher prevalence of haemorrhage patients.

Regarding the comparisons between ML and regression based models, our study also confirmed that there is only a small performance benefit of machine learning over carefully developed logistic regression models for clinical prediction models, which was consistent with prior literature[5,7]. The role of machine learning in quality improvement has not been extensively explored, but this study suggests that ML could have a role as part of the analysis and feedback of data about healthcare quality.

For peer review only - http://bmjopen.bmj.com/site/about/guidelines.xhtml

Strengths and limitations

The models were built and externally validated with very large and comprehensive stroke datasets (including data on 488,497 patients for development and validation and 128,360 patients for the external validation). Both of the data sources include a nearly complete population of hospitalised stroke in England and Wales (SSNAP) and Sweden (Riks-Stroke) reducing the risk of bias.

The models were built with robust approaches and reported according to the TRIPOD reporting guidelines with several terms adjusted for ML studies such as hyperparameter turning and final model presentation. The models were published and a have been made freely available for download for future external validation and use (GitHub repository: https://github.com/WenjuanW/Risk_Prediction_of_Post-Stroke_30-day_Mortality_SSNAP_and_RiksStroke).

The main limitations are: 1) Death outcomes were limited to inpatient mortality and it was not possible to ascertain deaths occurring outside hospital within 30 days for SSNAP data. But from the sensitivity analysis using all mortality from Riksstroke data, the rate of out-hospital mortality is small (2.3%) and the performance of external validation was good for both in-hospital mortality and all mortality. 2) NIHSS as a main risk predictor for mortality was not available in Riksstroke. Level of consciousness was used instead and according to previous studies Bray et al. 2014[6], the level of consciousness component of the NIHSS provides a good approximation of the full NIHSS for mortality prediction in settings where the ability to record the full NIHSS on admission is limited. 3) Some other risk factors are not available such as imaging, congestive heart failure etc. 4) The variables were limited to the ones that are available in both SSNAP and Riksstroke. However, all variables are generally available or can be derived from available variables in stroke registries which can benefit from the models developed in this study. 5) As can be expected, there were some discrepancies in how the included variables were recorded in the two registers. Well constructed methods were applied to handle the discrepancies and further sensitivity analysis were conducted. 6) Only XGboost was used for developing the model from ML-based methods but as we know that generally ML methods are not outperforming statistical methods with structured data, more ML methods would not be likely to add more information due to the limited number of variables.

For peer review only - http://bmjopen.bmj.com/site/about/guidelines.xhtml

Conclusions

We have developed and externally validated a machine learning based model to predict 30 day mortality after stroke, a widely collected and reported measure of stroke quality. The development of these types of generalisable models are important in allowing for unbiased comparisons of stroke outcomes across countries, and would be well suited to support efforts to measure and compare stroke outcomes as part of quality improvement initiatives.

Declarations

Ethics approval and consent to participate

SSNAP has approval from the Clinical Advisory Group of the NHS Health Research Authority to collect patient-level data under section 251 of the NHS Act 2006. No additional ethical review was sought. Informed consent was not sought for the present study because Data analyses were carried out using fully anonymised datasets from SSNAP.

The external validation using Riksstroke was approved by the Swedish Ethical Review Authority (reference no. 2021-06152-01). In accordance with the Personal Data Act (Swedish law No. SFS 1998:204), no informed consent is needed to collect data from medical charts and inpatient records for quality registers.

Data sharing statement

The data that support the findings of this study are available from SSNAP (www.strokeaudit.org) and Riksstroke (www.riksstroke.org) but restrictions apply to the availability of these data, which were used under license for the current study, and so are not publicly available. Data are however available from the SSNAP and Riksstroke upon reasonable request and with permission of HQIP and Riksstroke, respectively.

Competing interests

The authors declare that they have no competing interests

For peer review only - http://bmjopen.bmj.com/site/about/guidelines.xhtml

Funding

CDW, VC, and WW acknowledge the financial support from the Health Foundation. CDW acknowledge support from the National Institute for Health Research (NIHR) Biomedical Research Centre (BRC) based at Guy's and St Thomas' National Health Service (NHS) Foundation Trust and King's College London, and the NIHR Collaboration for Leadership in Applied Health Research and Care (ARC) South London at King's College Hospital NHS Foundation Trust. VC is supported by the National Institute for Health Research (NIHR) Biomedical Research Centre based at Guy's and St Thomas' National Health Service Foundation Trust and King's College London, and the Public Health and Multi-morbidity Theme of the National Institute for Health Research's Applied Research Collaboration (ARC) South London. VC is also supported by the EPSRC CONSULT grant (EP/P010105/1). The views expressed are those of the authors and not necessarily those of the NHS, the BRC or ARC.

Authors' contributions

Study design: WW, ME, BB

Literature review: WW, JO, ME, BB

Figures: WW, JO

Data cleaning and preprocessing: WW, JO

Model developing and internal validation (UK): WW, BB

External validation (Sweden): JO, ME

Data interpretation: WW, JO, ME, BB, CW, VC

Writing - original draft: WW, JO

Writing - review & editing: WW, BB, ME, JO, CW, VC

Supervision: BB (UK), ME (Sweden)

Funding acquisition (UK): BB, CW, VC

For peer review only - http://bmjopen.bmj.com/site/about/guidelines.xhtml

Project administration (UK): WW, BB, VC

Patient and Public Involvement

No patient involved

References

1. Jarman B, Gault S, Alves B, et al. Explaining differences in English hospital death rates using routinely collected data. *BMJ*. 1999;318(7197):1515-1520. doi:10.1136/bmj.318.7197.1515
2. Cadilhac DA, Kim J, Lannin NA, et al. National stroke registries for monitoring and improving the quality of hospital care: A systematic review. *Int J Stroke Off J Int Stroke Soc*. 2016;11(1):28-40. doi:10.1177/1747493015607523
3. Wang W, Kiik M, Peek N, et al. A systematic review of machine learning models for predicting outcomes of stroke with structured data. *PLOS ONE*. 2020;15(6):e0234722. doi:10.1371/journal.pone.0234722
4. Shah P, Kendall F, Khozin S, et al. Artificial intelligence and machine learning in clinical development: a translational perspective. *Npj Digit Med*. 2019;2(1):1-5. doi:10.1038/s41746-019-0148-3
5. Christodoulou E, Ma J, Collins GS, Steyerberg EW, Verbakel JY, Van Calster B. A systematic review shows no performance benefit of machine learning over logistic regression for clinical prediction models. *J Clin Epidemiol*. 2019;110:12-22. doi:10.1016/j.jclinepi.2019.02.004
6. Bray BD, Campbell J, Cloud GC, et al. Derivation and external validation of a case mix model for the standardized reporting of 30-day stroke mortality rates. *Stroke*. 2014;45(11):3374-3380. doi:10.1161/STROKEAHA.114.006451
7. Wang W, Rudd AG, Wang Y, et al. Risk prediction of 30-day mortality after stroke using machine learning: a nationwide registry-based cohort study. *BMC Neurol*. 2022;22(1):195. doi:10.1186/s12883-022-02722-1
8. O'Donnell MJ, Fang J, D'Uva C, et al. The PLAN score: a bedside prediction rule for death and severe disability following acute ischemic stroke. *Arch Intern Med*. 2012;172(20):1548-1556. doi:10.1001/2013.jamainternmed.30
9. Saposnik G, Kapral MK, Liu Y, et al. IScore: a risk score to predict death early after hospitalization for an acute ischemic stroke. *Circulation*. 2011;123(7):739-749. doi:10.1161/CIRCULATIONAHA.110.983353
10. Collins GS, Reitsma JB, Altman DG, Moons KG. Transparent reporting of a multivariable prediction model for individual prognosis or diagnosis (TRIPOD): the TRIPOD Statement. *BMC Med*. 2015;13(1):1. doi:10.1186/s12916-014-0241-z
11. Asplund K, Hulter Åsberg K, Appelros P, et al. The Riks-Stroke story: building a sustainable national register for quality assessment of stroke care. *Int J Stroke Off J Int Stroke Soc*. 2011;6(2):99-108. doi:10.1111/j.1747-4949.2010.00557.x
12. Fonarow GC, Saver JL, Smith EE, et al. Relationship of national institutes of health stroke scale to 30-day mortality in medicare beneficiaries with acute ischemic stroke. *J Am Heart Assoc*. 2012;1(1):42-50. doi:10.1161/JAHA.111.000034
13. Eriksson M, Appelros P, Norrving B, Terént A, Stegmayr B. Assessment of functional outcome in a national quality register for acute stroke: can simple self-reported items be transformed into the modified Rankin Scale? *Stroke*. 2007;38(4):1384-1386.

For peer review only - http://bmjopen.bmj.com/site/about/guidelines.xhtml

doi:10.1161/01.STR.0000260102.97954.9c
14. Azur MJ, Stuart EA, Frangakis C, Leaf PJ. Multiple imputation by chained equations: what is it and how does it work? *Int J Methods Psychiatr Res*. 2011;20(1):40-49. doi:10.1002/mpr.329
15. Zou H, Hastie T. Regularization and variable selection via the elastic net. *J R Stat Soc Ser B Stat Methodol*. 2005;67(2):301-320. doi:10.1111/j.1467-9868.2005.00503.x
16. Chen T, Guestrin C. XGBoost: A Scalable Tree Boosting System. In: *Proceedings of the 22nd ACM SIGKDD International Conference on Knowledge Discovery and Data Mining*. KDD '16. Association for Computing Machinery; 2016:785-794. doi:10.1145/2939672.2939785
17. Gerds TA, Cai T, Schumacher M. The performance of risk prediction models. *Biom J Biom Z*. 2008;50(4):457-479. doi:10.1002/bimj.200810443
18. Steyerberg EW, Vergouwe Y. Towards better clinical prediction models: seven steps for development and an ABCD for validation. *Eur Heart J*. 2014;35(29):1925-1931. doi:10.1093/eurheartj/ehu207
19. Van Calster B, McLernon DJ, van Smeden M, et al. Calibration: the Achilles heel of predictive analytics. *BMC Med*. 2019;17(1):230. doi:10.1186/s12916-019-1466-7
20. Vickers AJ, Elkin EB. Decision curve analysis: a novel method for evaluating prediction models. *Med Decis Mak Int J Soc Med Decis Mak*. 2006;26(6):565-574. doi:10.1177/0272989X06295361
21. Organised inpatient (stroke unit) care. doi:10.1002/14651858.CD000197.pub4

For peer review only

For peer review only - http://bmjopen.bmj.com/site/about/guidelines.xhtml

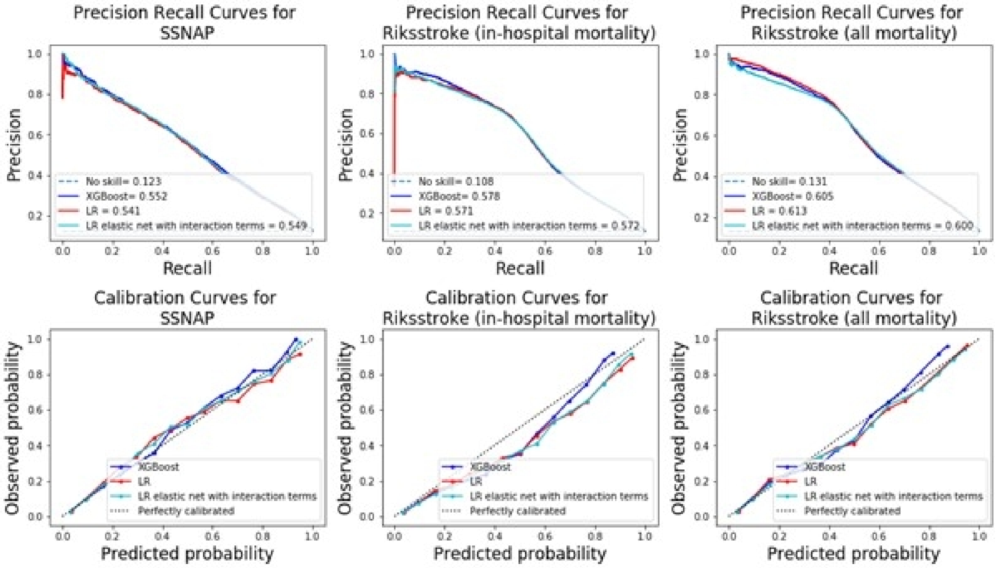

159x91mm (96 x 96 DPI)

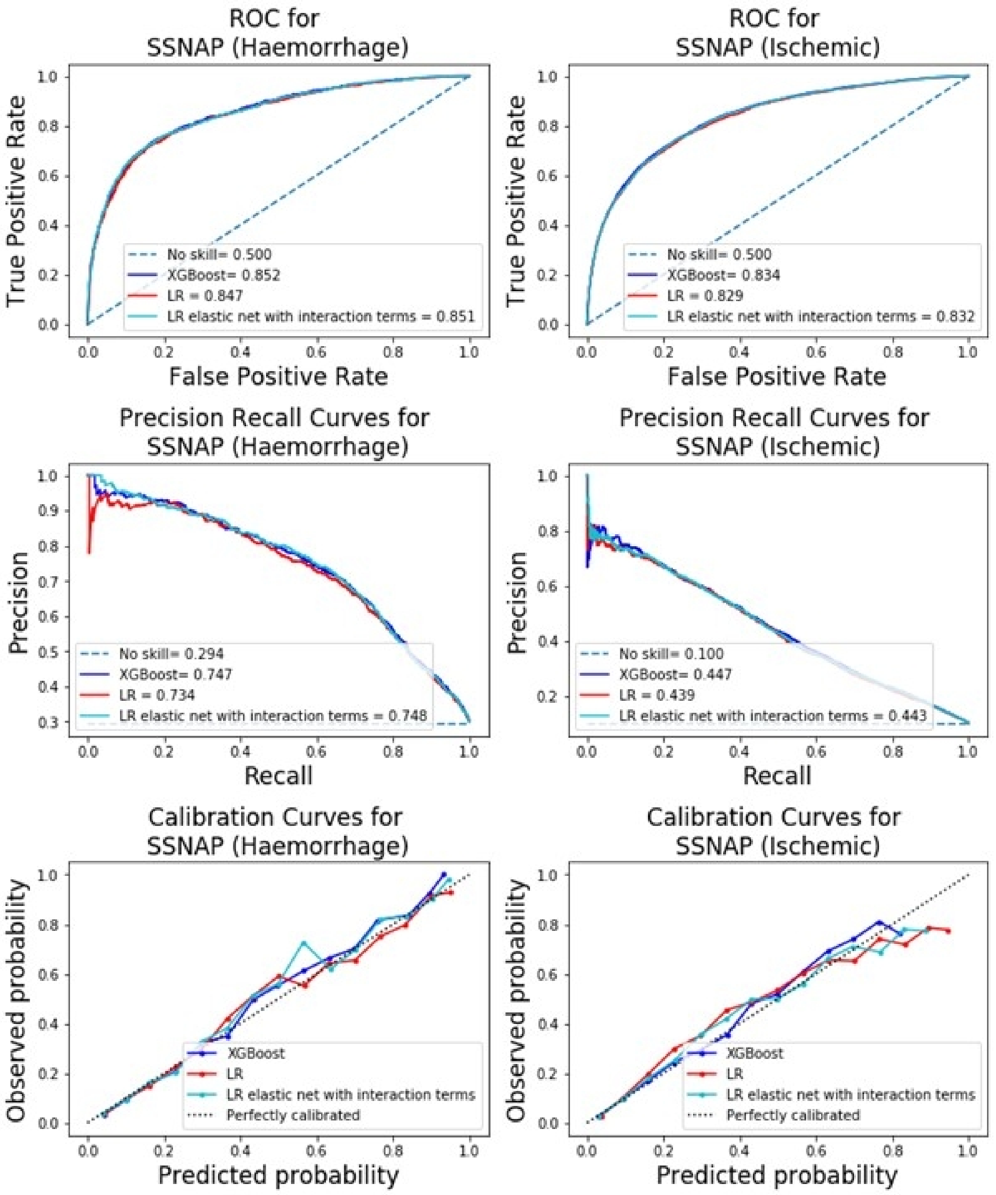

159x190mm (96 x 96 DPI)

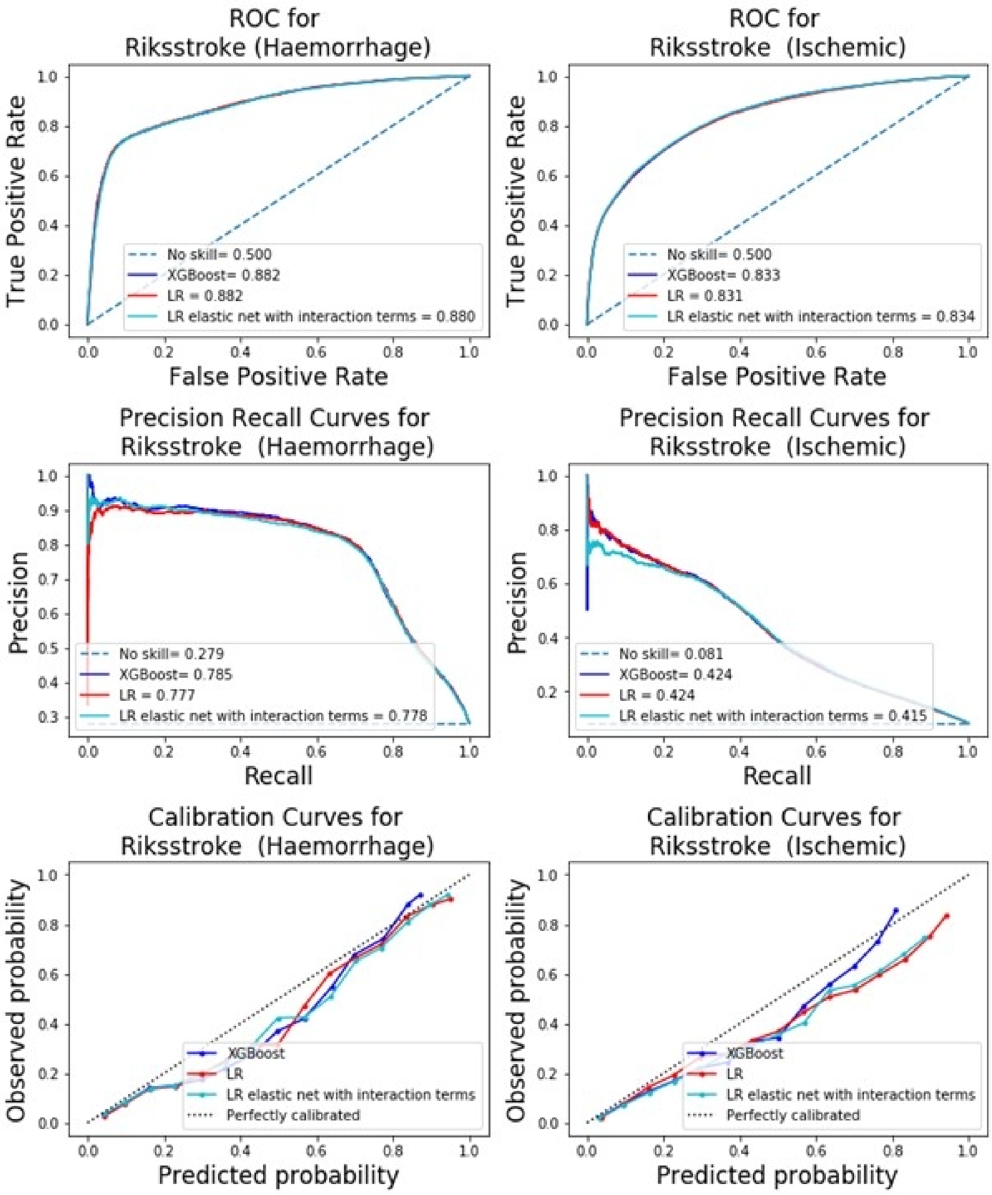

159x190mm (96 x 96 DPI)

Supplementary Material

Table A, Missing values in the variables and the imputation methods

	SSNAP		Riksstroke (in-hospital mortality)	
	**Missing (%)**	**Imputation method**	**Missing (%)**	**Imputation method**
Age groups	-	-	-	-
Male	-	-	-	-
Hypertension	-	-	0.5%	Multiple imputation
AF	-	-	0.2%	Multiple imputation
Diabetes	-	-	0.3%	Multiple imputation
Previous Stroke or TIA	-	-	0.3%	Multiple imputation
NIHSS at arrival	-	-	42.9%	Not used in the model
Level of consciousness	-	-	1.5%	Multiple imputation after splitting the non alert level to two levels
Pre-stroke mRS			5.0%	multiple imputation

For peer review only

anticoagulation prior to stroke if AF	58.9%	Unknown as a new category	-	-
Type of stroke	0.7%	Unknown as a new category	-	-

Abbreviation: AF: Atrial Fibrillation; TIA: transient ischaemic attack; NIHSS: National Institutes of Health Stroke Scale; mRS: modified Rankin Scale.

Table B: Brier score, AUC with 95% Confidence Interval (CI) for SSNAP development set and internal validation set.

	Brier score (95% CI)		AUC (95% CI)	
	SSNAP development set	SSNAP validation set	SSNAP development set	SSNAP validation set
LR	0.081 (0.080, 0.082)	0.079 (0.78, 0.81)	0.843 (0.841, 0.845)	0.845 (0.841, 0.849)
LR with elastic net and interaction terms	0.080 (0.079, 0.081)	0.078 (0.77, 0.80)	0.845 (0.843, 0.847)	0.848 (0.844, 0.851)
XGBoost	0.079 (0.079, 0.080)	0.078 (0.77, 0.79)	0.848 (0.846, 0.850)	0.849 (0.845, 0.853)

For peer review only

Table C, Brier score, AUC with 95% Confidence Interval (CI) for 2019 temporal validation and RiksStroke external validation with in-hospital mortality and all mortality.

	Brier score (95% CI)			AUC (95% CI)		
	SSNAP	RiksStroke (in-hospital mortality)	RiksStroke (all mortality)	SSNAP	RiksStroke (in-hospital mortality)	RiksStroke (all mortality)
LR	0.078 (0.076, 0.080)	0.066 (0.065, 0.067)	0.076 (0.075, 0.077)	0.847 (0.841, 0.853)	0.861 (0.857, 0.864)	0.859 (0.856, 0.862)
LR with elastic net and interaction terms	0.078 (0.076, 0.080)	0.067 (0.066, 0.068)	0.077 (0.076, 0.078)	0.850 (0.844, 0.856)	0.862 (0.859, 0.866)	0.861 (0.858, 0.864)
XGBoost	0.077 (0.075 ,0.079)	0.066 (0.065, 0.067)	0.077 (0.076, 0.078)	0.852 (0.846, 0.858)	0.861 (0.858, 0.865)	0.860 (0.857, 0.863)

Table D, the coefficients of variables from logistic regression

**Variables**	**Coefficients**
**loss of consciousness**	**1.26**

For peer review only

**type of stroke Haemorrhage**	**0.92**
**Inpatient at time of stroke**	**0.47**
**atrial fibrillation**	**0.46**
**type of stroke Unknown**	**0.32**
**Age (Groups by 5)**	**0.22**
**hour of admission 04 to 08**	**0.17**
**previous stroke/TIA**	**-0.16**
**prestroke mRS**	**0.16**
**hour of admission 12 to 16**	**-0.14**
**hypertension**	**-0.12**
**Male**	**0.11**
**admission Friday**	**-0.10**
**diabetes**	**0.10**
**hour of admission 16 to 20**	**-0.09**
**admission Monday**	**-0.08**
**prior anticoagulation if AF Nobut**	**0.07**
**admission Tuesday**	**-0.05**
**admission Wednesday**	**-0.05**

For peer review only

**admission Thursday**	**-0.05**
**admission Saturday**	**-0.03**
**hour of admission 08 to 12**	**-0.03**
**prior anticoagulation if AF Yes**	**-0.02**
**hour of admission 20 to 24**	**0.02**
**prior anticoagulation if AF Unknown**	**-0.005**

Abbreviation: AF: Atrial Fibrillation; TIA: transient ischaemic attack; mRS: modified Rankin Scale.

For peer review only

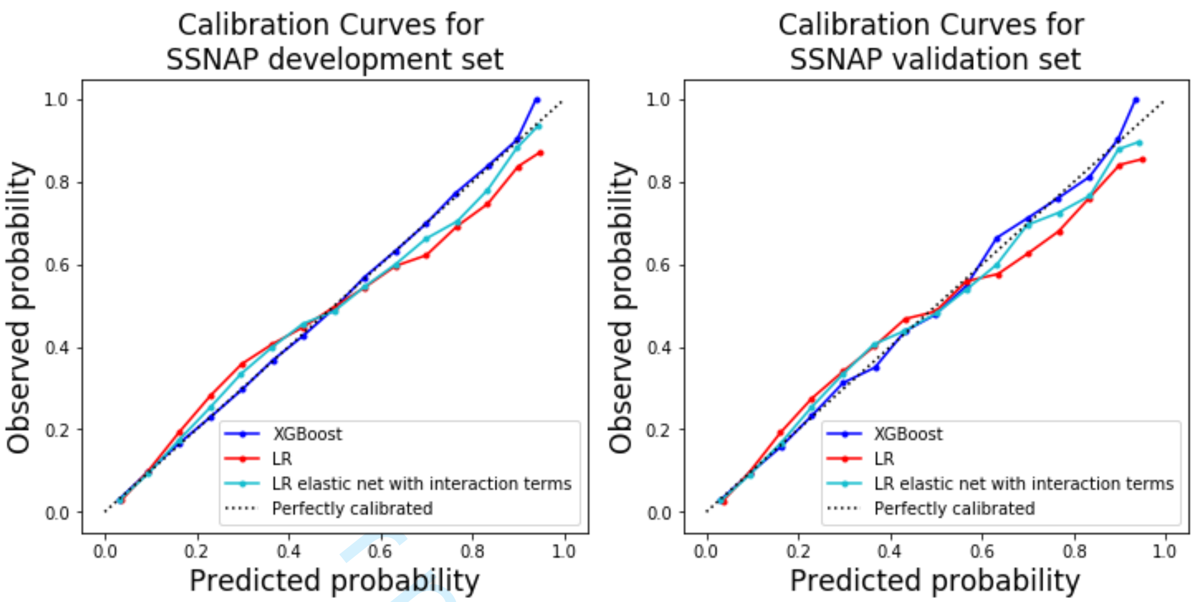

Figure A: Calibration curves for all models on development set and validation set of SSNAP cohort.

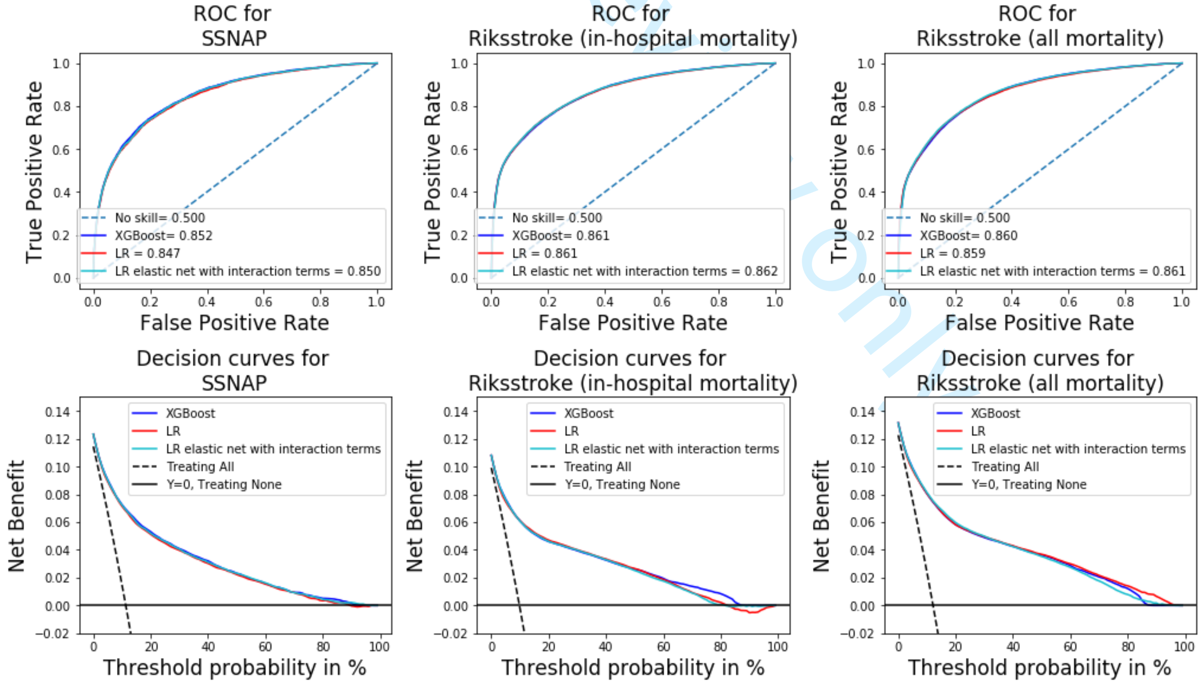

Figure B: ROC and decision curves for all models on 2019 temporal validation of SSNAP cohort (left) and external validation using Riksstroke with in-hospital mortality (middle) and all mortality (right)

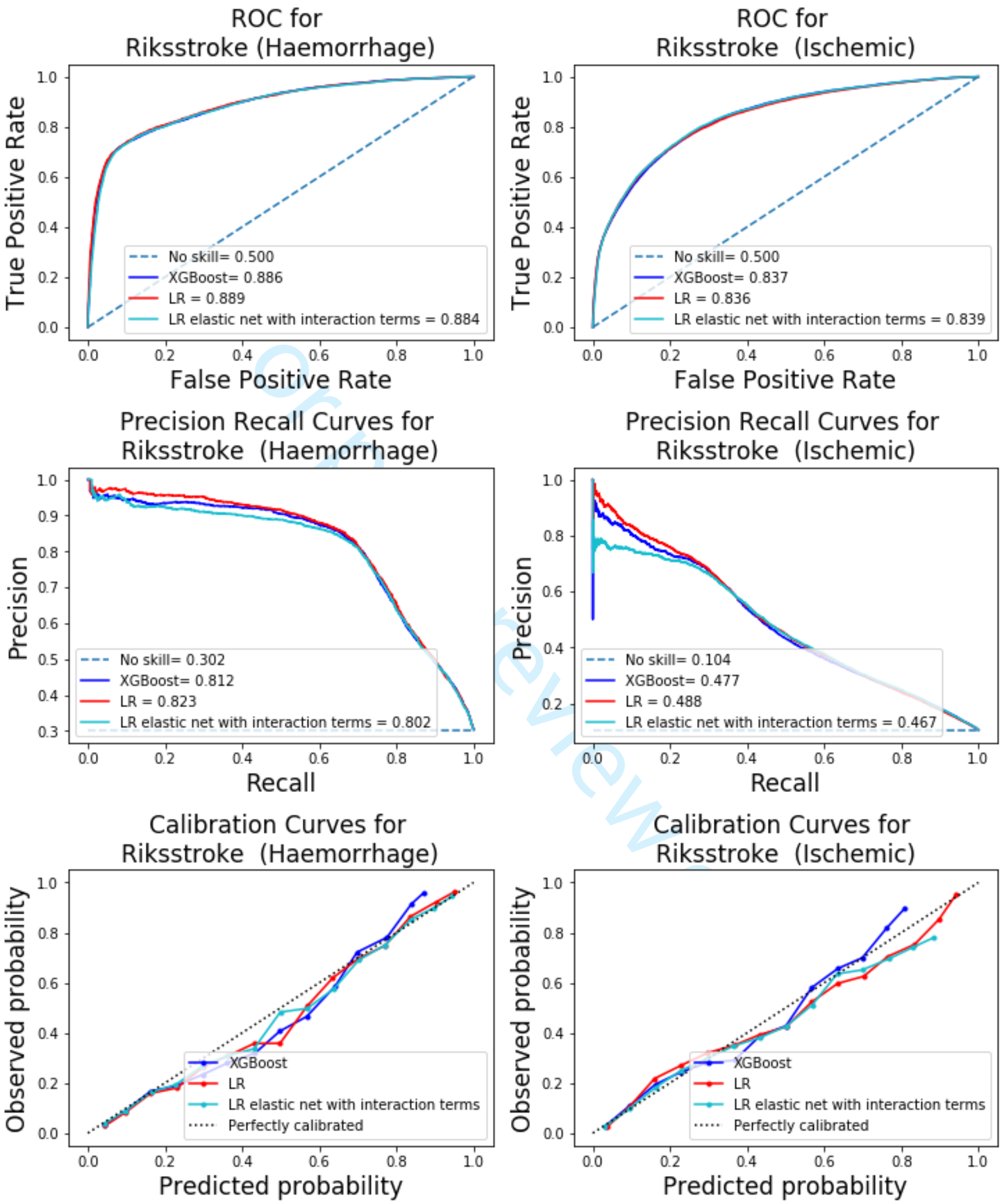

Figure C: AUC-ROC, PR-curve, and Calibration-curve for all mortalities (Haemorrhage (left) vs Ischemic (right) patients) in Riksstroke

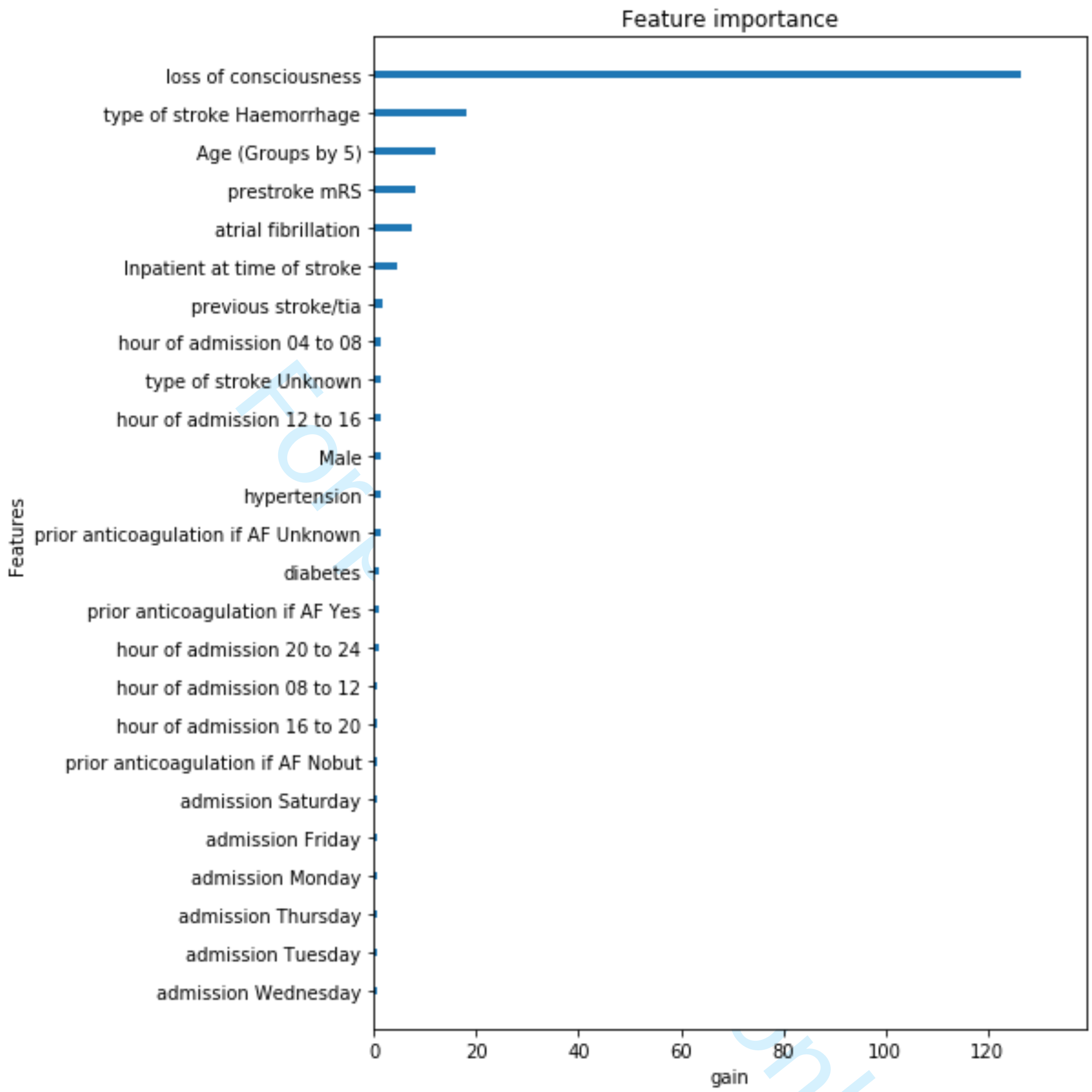

Figure D: Feature importance from XGBoost using gain as measurement of feature importance

Table: Transparent reporting of a multivariable prediction model for individual prognosis or diagnosis (TRIPOD) checklist

Section/ Topic	m	Checklist Item	Page
**Title and abstract**			
Title	1	Identify the study as developing and/or validating a multivariable prediction model, the target population, and the outcome to be predicted.	0
Abstract	2	Provide a summary of objectives, study design, setting, participants, sample size, predictors, outcome, statistical analysis, results, and conclusions.	1
**Introduction**			
Background and objectives	3a	Explain the medical context (including whether diagnostic or prognostic) and rationale for developing or validating the multivariable prediction model, including references to existing models.	4
	3b	Specify the objectives, including whether the study describes the development or validation of the model or both.	4-5
**Methods**			
Source of data	4a	Describe the study design or source of data (e.g., randomized trial, cohort, or registry data), separately for the development and validation data sets, if applicable.	5-6

For peer review only - http://bmjopen.bmj.com/site/about/guidelines.xhtml

	4b	Specify the key study dates, including start of accrual; end of accrual; and, if applicable, end of follow-up.	5--6
Participants	5a	Specify key elements of the study setting (e.g., primary care, secondary care, general population) including number and location of centres.	5
	5b	Describe eligibility criteria for participants.	5
	5c	Give details of treatments received, if relevant.	-
Outcome	6a	Clearly define the outcome that is predicted by the prediction model, including how and when assessed.	6
	6b	Report any actions to blind assessment of the outcome to be predicted.	-
Predictors	7a	Clearly define all predictors used in developing or validating the model, including how and when they were measured.	6-7
	7b	Report any actions to blind assessment of predictors for the outcome and other predictors.	-
Sample size	8	Explain how the study size was arrived at.	5
Missing data	9	Describe how missing data were handled (e.g., complete-case analysis, single imputation, multiple imputation) with details of any imputation method.	7
Statistical analysis	10a	Describe how predictors were handled in the analyses.	7
	10b	Specify type of model, all model-building procedures and method for internal validation.	7-8

For peer review only

s method s	10d	Specify all measures used to assess model performance and, if relevant, to compare multiple models.	8-9
Risk groups	11	Provide details on how risk groups were created, if done.	9
**Results**			
Particip ants	13a	Describe the flow of participants through the study, including the number of participants with and without the outcome and, if applicable, a summary of the follow-up time. A diagram may be helpful.	-
	13b	Describe the characteristics of the participants (basic demographics, clinical features, available predictors), including the number of participants with missing data for predictors and outcome.	9-10
Model develo pment	14a	Specify the number of participants and outcome events in each analysis.	9
	14b	If done, report the unadjusted association between each candidate predictor and outcome.	-
Model specific ation	15a	Present the full prediction model to allow predictions for individuals.	13
	15b	Explain how to the use the prediction model.	13
Model perfor mance	16	Report performance measures (with CIs) for the prediction model.	13-14
**Discussion**			

For peer review only - http://bmjopen.bmj.com/site/about/guidelines.xhtml

Limitations	18	Discuss any limitations of the study (such as nonrepresentative sample, few events per predictor, missing data).	16
Interpretation	19b	Give an overall interpretation of the results, considering objectives, limitations, and results from similar studies, and other relevant evidence.	15-16
Implications	20	Discuss the potential clinical use of the model and implications for future research.	15
**Other information**			
Supplementary information	21	Provide information about the availability of supplementary resources, such as study protocol, Web calculator, and data sets.	Yes
Funding	22	Give the source of funding and the role of the funders for the present study.	Yes

For peer review only

BMJ Open

Developing and externally validating a machine learning risk prediction model for 30-day mortality after stroke using national stroke registers in the UK and Sweden

Journal:	*BMJ Open*
Manuscript ID	bmjopen-2022-069811.R1
Article Type:	Original research
Date Submitted by the Author:	20-Jun-2023
Complete List of Authors:	Wang , Wenjuan; King's College London<br>Otieno, Josline; Umeå University, Department of Statistics<br>Eriksson, Marie; Umea Universitet, Statistics<br>Wolfe, Charles; King's College London<br>Curcin, Vasa; King's College London<br>Bray, Benjamin; King's College London
**Primary Subject Heading**:	Neurology
Secondary Subject Heading:	Neurology
Keywords:	Quality in health care < HEALTH SERVICES ADMINISTRATION & MANAGEMENT, NEUROLOGY, STATISTICS & RESEARCH METHODS, Stroke < NEUROLOGY

SCHOLARONE™ Manuscripts

I, the Submitting Author has the right to grant and does grant on behalf of all authors of the Work (as defined in the below author licence), an exclusive licence and/or a non-exclusive licence for contributions from authors who are: i) UK Crown employees; ii) where BMJ has agreed a CC-BY licence shall apply, and/or iii) in accordance with the terms applicable for US Federal Government officers or employees acting as part of their official duties; on a worldwide, perpetual, irrevocable, royalty-free basis to BMJ Publishing Group Ltd ("BMJ") its licensees and where the relevant Journal is co-owned by BMJ to the co-owners of the Journal, to publish the Work in this journal and any other BMJ products and to exploit all rights, as set out in our licence.

The Submitting Author accepts and understands that any supply made under these terms is made by BMJ to the Submitting Author unless you are acting as an employee on behalf of your employer or a postgraduate student of an affiliated institution which is paying any applicable article publishing charge ("APC") for Open Access articles. Where the Submitting Author wishes to make the Work available on an Open Access basis (and intends to pay the relevant APC), the terms of reuse of such Open Access shall be governed by a Creative Commons licence – details of these licences and which Creative Commons licence will apply to this Work are set out in our licence referred to above.

Other than as permitted in any relevant BMJ Author's Self Archiving Policies, I confirm this Work has not been accepted for publication elsewhere, is not being considered for publication elsewhere and does not duplicate material already published. I confirm all authors consent to publication of this Work and authorise the granting of this licence.

For peer review only

Developing and externally validating a machine learning risk prediction model for 30-day mortality after stroke using national stroke registers in the UK and Sweden

Wenjuan Wang[1], Josline Adhiambo Otieno[2], Marie Eriksson[2], Charles D Wolfe[1,3], Vasa Curcin[1,3], Benjamin D Bray[1]

1 School of Population Health and Environmental Sciences, King's College London, London, UK

2 Department of Statistics, Umeå School of Business, Economics and Statistics (USBE), Umeå University, Umeå, Sweden

3 NIHR Biomedical Research Centre, Guy's and St Thomas' NHS Foundation Trust

Corresponding author: Dr Wenjuan Wang, Addison House 3.07, Guy's Campus, London SE1 1UL, UK. Email: wenjuan.wang@kcl.ac.uk

Keywords: stroke, mortality, risk prediction, quality improvement, external validation

Word count: 4156

For peer review only - http://bmjopen.bmj.com/site/about/guidelines.xhtml

Abstract

Objectives

We aimed to develop and externally validate a generalisable risk prediction model for 30-day stroke mortality suitable for supporting quality improvement analytics in stroke care using large nationwide stroke registers in the United Kingdom and Sweden.

Design

Registry based cohort study.

Setting

Stroke registries including the Sentinel Stroke National Audit Program (SSNAP) in England, Wales, and Northern Ireland (2013-2019) and the national Swedish stroke register (Riksstroke 2015-2020).

Participants and Methods

Data from SSNAP were used for developing and temporally validating the model, and data from Riksstroke were used for external validation. Models were developed with the variables available in both registries using logistic regression (LR), LR with elastic net and interaction terms, and XGBoost. Performances were evaluated with discrimination, calibration, and decision curves.

Outcome measures

The primary outcome was all-cause 30-day in-hospital mortality after stroke.

Results

In total, 488,497 stroke patients with 12.4% 30-day in-hospital mortality were used for developing and temporally validating the model in the UK. A total of 128,360 stroke patients with 10.8% 30-day in-hospital mortality and 13.1% all mortality were used for external validation in Sweden. In the SSNAP temporal validation set, the final XGBoost model achieved the highest area under the ROC curve (AUC) (0.852 (95% CI: 0.848-0.855)) and was well-calibrated. The performances on the external validation in Riksstroke were as good and achieved AUC at 0.861 (95% CI: 0.858, 0.865) for in-hospital mortality. For Riksstroke, the

For peer review only - http://bmjopen.bmj.com/site/about/guidelines.xhtml

models slightly overestimated the risk for in-hospital mortality, whilst they were better calibrated at the risk for all mortality.

Conclusion

The risk prediction model was accurate and externally validated using high quality registry data. This is potentially suitable to be deployed as part of quality improvement analytics in stroke care to enable the fair comparison of stroke mortality outcomes across hospitals and health systems across countries.

Strengths and limitations of this study

- The models were built and externally validated with very large and comprehensive stroke datasets from a nearly complete population of hospitalised stroke in England and Wales (SSNAP) and Sweden (Riksstroke).
- The models were built with robust approaches and reported according to the TRIPOD reporting guidelines with several terms adjusted for ML studies such as hyperparameter tuning.
- Final models were published and have been made freely available for download for future external validation and use.
- Death outcomes were limited to inpatient mortality and was not possible to ascertain deaths occurring outside hospital within 30 days for SSNAP data.
- The predictors were limited to the variables that are available in both SSNAP and Riksstroke.

For peer review only - http://bmjopen.bmj.com/site/about/guidelines.xhtml

Introduction

Risk prediction models are valuable tools in benchmarking health care outcomes across different hospitals or countries, thereby contributing to improved clinical practice [1]. These models are required to adjust for differences in the case mix of patients to allow for an unbiased comparison of stroke outcomes and can be used to identify patients at high risk of poor outcomes. Benchmarking and comparing stroke outcomes across hospitals and health systems is widely used as part of quality improvement initiatives and quality registers in stroke [2], but depends on accurate adjustment of case mix differences to be valid.

Prediction models using machine learning (ML) algorithms are now being widely used in healthcare (e.g. in the interpretation of medical imaging data) but the potential of ML to improve the accuracy of analytics used for measuring and comparing the quality of stroke care services is largely unexplored. Moreover, a systematic review on the application of ML methods in predicting outcomes of stroke based on structured data found that few studies met basic reporting standards for clinical prediction tools [3]. Major issues included lack of external validation, and the non-availability of the final model for external validation [3]. External validation (i.e. evaluating the accuracy of the predictions in data from a different source that used to develop the model) is especially important in assessing the generalizability of the model before it is used in other settings.

Traditionally, logistic regression (LR) has been used for risk predictions of stroke outcome. It has been suggested that ML might outperform clinical prediction models based on LR because they make fewer assumptions and can learn complex relationships between predictors and outcomes [4]. However, there are also studies showing that no evidence of superior performance of ML over LR and improvements in methodology and reporting are needed for studies that compare modelling algorithms [5]. In a previous study, we compared updated LR models from Bray et al. 2014 [6] and a ML algorithm (XGBoost) for predicting 30-day mortality after stroke and found that XGBoost performed the best[7][7]. Existing scores for 30-day mortality prediction, e.g., PLAN [8] and iScore [9] were not compared in the previous study due to lack of certain variables.

Models developed and validated in one nation might not perform well with another nation due to the differences in the population and health systems. In this study, we aimed to develop and externally validate a generalisable risk prediction model for 30-day mortality after stroke using large nationwide stroke registers in the United Kingdom and Sweden. The intention was to

For peer review only - http://bmjopen.bmj.com/site/about/guidelines.xhtml

develop a model developed and externally validated in high quality registry data which could be made publicly available to support stroke research (e.g. cross national or multi-centre comparisons of stroke mortality) or be deployed as part of quality improvement analytics initiatives in stroke.

For peer review only

For peer review only - http://bmjopen.bmj.com/site/about/guidelines.xhtml

Methods

This study is reported according to the TRIPOD guidelines: transparent reporting of a multivariable prediction model for individual prognosis or diagnosis [10].

Data source

Data from SSNAP for model development and internal validation

Data for developing the models were from Sentinel Stroke National Audit Programme (SSNAP), the national registry of stroke care in England, Wales and Northern Ireland. SSNAP is a Healthcare Quality Improvement Partnership (HQIP) register for stroke care quality improvement. Data were collected prospectively, validated by clinical teams, and entered into the SSNAP database via a secure web interface. SSNAP includes an estimated 95% of all adults admitted to hospital with acute stroke (ischaemic or primary intracerebral haemorrhage) in England, Wales and Northern Ireland. The original dataset from SSNAP was collected from 333 teams across England, Wales and Northern Ireland between April 1, 2013 to June 30, 2019. For the generalisation of the model to the population, all patients were included and no specific exclusion criteria for stroke patients.

Data from Riksstroke for external validation

We then used data from the Swedish stroke register (Riksstroke) for external validation of the developed models. Riksstroke is a national quality register, monitoring stroke care and outcome in adult patients. Details about the register and included variables have been published previously [11] and the forms can be found on Riksstroke's webpage (https://www.riksstroke.org/forms/).

In short, all hospitals admitting acute stroke (ischaemic, primary intracerebral haemorrhage, or unspecified type of stroke) patients in Sweden participate (currently 72 hospitals). Riksstroke is estimated to include more than 90% of all acute stroke patients treated in hospital. The register includes information from the entire chain of stroke care, including primary prevention, acute management, rehabilitation, secondary prevention, and family and community support. The dataset for external validation included patients who experienced stroke between January 1, 2015 to December 31, 2020.

Availability of data and materials

For peer review only - http://bmjopen.bmj.com/site/about/guidelines.xhtml

The data that support the findings of this study are available from SSNAP (www.strokeaudit.org) and Riksstroke (www.riksstroke.org) but restrictions apply to the availability of these data, which were used under license for the current study, and so are not publicly available. Data are however available from the SSNAP and Riksstroke upon reasonable request and with permission of HQIP and Riksstroke, respectively.

Study outcome

For models developed with data from the SSNAP registry, the outcome was all-cause 30-day in-hospital mortality post-stroke. All patients had in-hospital status due to the collection procedure in SSNAP. Out-hospital deaths were not available for analysis.

For the external validation with data from Riksstroke, date of death was retrieved for all patients by individual linkage with the national Cause of Death register, managed by the National Board of Health and Welfare, using personal identification numbers. Hence, all 30-day mortality included both in- and out-of-hospital deaths.

The developed models with SSNAP data were externally validated with both Riksstroke in-hospital deaths and all deaths as outcomes. Performances for in-hospital death were compared between SSNAP and Riksstroke. Comparisons between all deaths and in-hospital death in Riksstroke were conducted as a sensitivity analysis.

Predictor variables

In total, 65 variables were available from SSNAP. Details on how each risk predictor was measured have been presented in a previous study for variables in SSNAP [7] and a previous study for variables in Riksstroke [11]. Subsequently, 13 variables were used to build and validate prediction models of 30-day mortality. The variable selection was done based on the clinical importance, literature review, and availability in both the SSNAP and Riksstroke registries. Different clinical covariates for prediction included age (band by 5), sex, inpatient at time of stroke, hour of admission, day of week of admission, comorbidities such as hypertension, AF, diabetes, previous stroke or transient ischaemic attack (TIA), and anticoagulation prior to stroke if AF, pre-stroke modified Rankin Scale (mRS), loss of consciousness, and type of stroke (ischemic or primary intracerebral haemorrhage). The National Institutes of Health Stroke Scale (NIHSS) was not used as a variable/predictor due to the high missingness in Riksstroke (42.9%). Bray et al. 2014 showed that the level of

For peer review only - http://bmjopen.bmj.com/site/about/guidelines.xhtml

consciousness component of the NIHSS provides a good approximation of the full NIHSS for mortality prediction [2].

Two predictors were recorded differently in SSNAP and Riksstroke, i.e. level of consciousness and pre-stroke mRS. In SSNAP, level of consciousness was assessed based on the first item of the NIHSS which has four levels, i.e., 0 = alert, 1 = not alert but arousable by minor stimulation, 2 = not alert and requires repeated stimulation to attend, and 3 = unconscious. In Riksstroke, level of consciousness was recorded in three categories where alert corresponds to level of consciousness 0 in SSNAP, not alert to level of consciousness 1-2 in SSNAP, and unconscious level of consciousness 3 in SSNAP. To obtain 4 categories in Riksstroke, a total NIHSS score ≤13 vs. >13 was used to split the mid category (not alert). The cutoff was chosen based on the NIHSS score distribution in Riksstroke and previous studies showing good discrimination properties for 30-day stroke mortality [12]. In Riksstroke pre-stroke mRS was estimated using a previously developed algorithm [13] which has four categories with the first category corresponding to 0-2 and the next three categories corresponding to 3, 4, 5 in the pre-Stroke mRS in SSNAP. The first category was imputed to be 0 in Riksstroke for the external validation.

Missing data

Missing data were handled using different methods according to assumptions of missing mechanism after consulting the SSNAP team and clinicians. Variables with more than 80% missingness were discarded. For categorical variables with missing by design/not applicable assumption, missing values were added as a new category. Missing indicator was used for missing by design/not applicable continuous variables. After these, Multiple Imputation with Chained Equations (MICE) [14] was used to impute variables with missing at random assumption for the development dataset. While the missing mechanism is impossible to prove, MICE is seen as a better choice according to several references when comparing it with different imputation methods [15]. All available variables except for the discarded ones were used in MICE. Five datasets were imputed using MICE and aggregated into one using the median for SSNAP with all the variables available in the dataset and Riksstroke dataset. Details for handling missing data in SSNAP were presented in a previous study [7].

Model building and evaluation

For peer review only - http://bmjopen.bmj.com/site/about/guidelines.xhtml

After variable selection and imputation of missing values, the variables were pre-processed. Details of preprocessing and coding of variables were presented in a previous study [7] and in the GitHub repository (https://github.com/WenjuanW/Risk_Prediction_of_Post-Stroke_30-day_Mortality_SSNAP_and_RiksStroke). Descriptive statistics were used to compare the characteristic of death/alive at 30-day across the entire datasets and between SSNAP and Riksstroke.

Models were developed/trained with data from SSNAP using 80% randomly selected patients from 2013 to 2018, validated on the remaining 20% data from 2013 to 2018, and temporally validated on data from 2019. Subsequently, models were externally validated using data from Riksstroke from between January 1, 2015 to December 31, 2020.

Due to the lack of final models from other ML studies and lack of certain variables for risk scores (PLAN score [8]: cancer, Iscore [9]: Cancer, renal dialysis, stroke scale score, non-lacunar, and abnormal glucose level), we could not externally validate existing ML models and risk scores. In previous studies comparing the performance of Logistic regression (LR) models with ML models, models developed using XGBoost generally performed the best [7]. Thus, new models were built with LR, LR with elastic net [16] with interaction terms, and eXtreme Gradient Boosting (XGBoost) [17] using the mutual risk predictors that were available in both countries in order to externally validate the models. Best hyperparameters (a parameter that is predefined by the user to control the learning process) were selected on the development set with grid search or random search and cross-validation (CV). Detailed hyperparameter tuning strategy was presented in a repository on Github that was built for this study (https://github.com/WenjuanW/Risk_Prediction_of_Post-Stroke_30-day_Mortality_SSNAP_and_RiksStroke).

Brier score [18] was used as an overall summative measure of predictive performance. Discrimination was measured by area under the ROC curve (AUC). Calibration was visually assessed with calibration plots [19] rather than Hosmer-Lemeshow (HL) test as HL test has a number of drawbacks, including limited power, poor interpretability and is not recommended [19]. Python functions for calculating these measurements were presented in the Github repository.

Comparisons of AUCs and Brier scores within each set were conducted with Delong's test and paired t-test respectively. To compare AUCs between the performances on SSNAP and

For peer review only - http://bmjopen.bmj.com/site/about/guidelines.xhtml

Riksstroke, an independent group t-test was used for both AUCs and Brier scores with 500 bootstraps for each measurement.

Due to the low outcome rate, the dataset is imbalanced at predicting the outcomes. In this case, AUC cannot fully reflect the performances of the models on positive predictive rate (precision) and true positive rate (recall). Therefore, precision and recall were assessed via area under the precision-recall curves and the precision-recall curves.

Clinical utility was assessed with decision curve analysis [20] which graphically shows the net benefit obtained by applying the strategy of treating an individual if and only if predicted risk is larger than a threshold in function of the threshold probability. Threshold equals to 0 means treating all since all predicted risk will be larger than 0. Threshold equals to 1 means treating none since all predicted risk will be smaller than 1. All analyses were conducted using Python 3.7.4.

As stroke type is a critical factor for outcomes of stroke patients, we subsequently performed a subgroup analysis to investigate the performance of the developed models for patients with different stroke types i.e. infarction and primary intracerebral haemorrhage.

Sensitivity analysis

External validation on all 30-day mortality (i.e including deaths outside of hospital) in Riksstroke was conducted as a sensitivity analysis to check the impact of using only in-hospital mortality for developing the models. Another sensitivity analysis was done using mRS = 1 as the level representing 0-2 in previous stroke mRS variable in Riksstroke.

Patient and Public Involvement

Neither the patients nor the public were involved in this study.

For peer review only

For peer review only - http://bmjopen.bmj.com/site/about/guidelines.xhtml

Results

Participants

The characteristics of 488,947 patients from SSNAP and 128,360 patients from Riksstroke were reported in Table 1. In total, 60,362 (12.4%) patients from SSNAP and 13,873 (10.8%) from Riksstroke died within 30 days in hospital and (13.1% for all mortality within 30 days). In both SSNAP and Riksstroke, patients who died within 30 days were older, and were more likely to have cardiovascular risk factors, impaired functional status prior to stroke, haemorrhagic stroke, or being unconscious upon arrival to hospital (Table 1).

The patient case-mix in SSNAP and Riksstroke was similar with respect to age distribution. Riksstroke had a high percentage of males for patients who died at 30 days. A higher frequency in Riksstroke compared to SSNAP was observed in hypertension (63.0% vs 54.2%), AF (28.9% vs 19.7%), diabetes (22.4% bs 20.9%), previous stroke or TIA (27.4% vs 26.5%), and haemorrhage (13.1% vs 11.5%). The difference in these risk factors is even larger for the patients who died with 30-days after stroke with hypertension (67.4% vs 53.5%), AF (43.5% vs 33.0%), diabetes (22.6% bs 20.2%), previous stroke or TIA (31.6% vs 28.3%), and haemorrhagic stroke (33.8% vs 27.2%). A lower percentage of high level of consciousness (level 3) was observed in Riksstroke compared to SSNAP (4.6% vs 6.8%). On the contrary, a higher percentage of high levels of previous stroke mRS was found in Riksstroke compared to SSNAP (2.82% vs 1.8% for level 5, 9.2% vs 6.2% for level 4, and 14.8% vs 11.7% for level 3).

Summary of missing percentages and imputation methods for all predictors were presented in Supplementary Table A. In SSNAP, only the type of stroke predictor had missing values of 0.7% and the missing values were taken as a new category. In Riksstroke, pre-stroke mRS had 5.0% missing values and level of consciousness had 1.5% missing values. Missing percentages for comorbidity variables were small (less than 0.5%).

For peer review only - http://bmjopen.bmj.com/site/about/guidelines.xhtml

Table 1, Patients characteristics in SSNAP and Riksstroke

	SSNAP			Riksstroke (in-hospital mortality)		
	**Overall**	**Alive at 30 days**	**Dead at 30 days**	**Overall**	**Alive at 30 days**	**Dead at 30 days**
No. cases (%)	488,947	428,585 (87.6)	60,362 (12.4)	128,360	114,487 (89.2)	13,873 (10.8)
Age 15-60 (%)	76,941 (15.7)	74,238 (17.3)	2,703 (4.5)	15,586 (12.1)	14,958 (13.1)	628 (4.5)
Age 61-70 (%)	84,484 (17.3)	79,109 (18.5)	5,375 (8.9)	23,186 (18.1)	21,782 (19.0)	1,404 (10.1)
Age 71-80 (%)	136,728 (28.0)	122,393 (28.6)	14,335 (23.7)	41,396 (32.3)	37,689 (32.9)	3,707 (26.7)
Age 81+ (%)	190,794 (39.0)	152,845 (35.7)	37,949 (62.9)	48,192(37.5)	40,058 (35.0)	8,134 (58.6)
Male (%)	249,291 (51.0)	223,849 (52.2)	25,442 (42.1)	68,985 (53.7)	62,428 (54.5)	6,557 (47.3)
Hypertension (%)	264,806 (54.2)	232,505 (54.2)	32,301 (53.5)	80,824 (63.0)	71,481 (62.4)	9,343 (67.4)
AF (%)	96,354 (19.7)	76,417 (17.8)	19,937 (33.0)	37,090 (28.9)	31,057 (27.1)	6,033(43.5)
Diabetes (%)	102,324 (20.9)	90,104 (21.0)	12,220 (20.2)	28,722 (22.4)	25,589 (22.4)	3,133 (22.6)
Previous Stroke or TIA (%)	129,462 (26.5)	112,358 (26.2)	17,104 (28.3)	35,145 (27.4)	30,757 (26.9)	4,388 (31.6)
NIHSS at arrival (median [IQR])	4.00 [2.0, 10.0]	4.00 [2.0, 8.0]	17.00 [7.0, 23.0]	3.00 [1.0, 8.0]	3.00 [1.0, 7.0]	16.00 [9.0, 23.0]
Level of consciousness (%)*						
0 (alert)	391,065 (80.0)	379,085 (88.5)	11,980 (19.8)	106,474 (83.0)	101,706 (88.8)	4,768 (34.4)
1 (not alert)	419,16 (8.6)	32,715 (7.6)	9,201 (15.2)	14,067 (11.0)	9,459 (8.3)	4,608 (33.2)
2 (not alert)	22,635 (4.6)	11,790 (2.8)	10,845 (18.0)			
3 (unconscious)	33,331 (6.8)	4,995 (1.2)	28,336 (46.9)	5,930 (4.6)	1,759 (1.5)	4,171 (30.1)
Pre-stroke mRS						

For peer review only

<table>
<tr><th></th><th colspan="3">SSNAP</th><th colspan="3">Riksstroke (in-hospital mortality)</th></tr>
<tr><td>(%)</td><td></td><td></td><td></td><td></td><td></td><td></td></tr>
<tr><td>0</td><td>266,409 (54.5)</td><td>245,979 (57.4)</td><td>20,430 (33.8)</td><td rowspan="3">87,593 (68.2)</td><td rowspan="3">81,871 (71.5)</td><td rowspan="3">5,722 (41.3)</td></tr>
<tr><td>1</td><td>75,661 (15.5)</td><td>66,940 (15.6)</td><td>8,721 (14.4)</td></tr>
<tr><td>2</td><td>50,795 (10.4)</td><td>42,592 (9.9)</td><td>8,203 (13.6)</td></tr>
<tr><td>3</td><td>56,994 (11.7)</td><td>44,601 (10.4)</td><td>12,393 (20.5)</td><td>18,942 (14.8)</td><td>16,563(14.5)</td><td>2,379 (17.2)</td></tr>
<tr><td>4</td><td>30,276 (6.2)</td><td>22,339 (5.2)</td><td>7,937 (13.1)</td><td>11,763 (9.2)</td><td>9,483 (8.3)</td><td>2,280 (16.4)</td></tr>
<tr><td>5</td><td>8,812 (1.8)</td><td>6,134 (1.4)</td><td>2,678 (4.4)</td><td>3,623 (2.8)</td><td>2,720 (2.4)</td><td>903 (6.5)</td></tr>
<tr><td>Haemorrhage (%)</td><td>55,758 (11.5)</td><td>39,472 (9.3)</td><td>16,286 (27.2)</td><td>16,757 (13.1)</td><td>12,074 (10.6)</td><td>4,683 (33.8)</td></tr>
</table>

Abbreviation: AF: Atrial Fibrillation; TIA: transient ischaemic attack; NIHSS: National Institutes of Health Stroke Scale; mRS: modified Rankin Scale. * Level of consciousness is measured differently between SSNAP. In SSNAP, Level of consciousness has four levels (0 = alert, 1 = not alert but arousable by minor stimulation, 2 = not alert and requires repeated stimulation to attend, and 3 = unconscious). In Riksstroke, Level of consciousness has three levels (0 = alert, 1,2 = not alert, 3 = unconscious).

Table 2, Brier score, AUC with 95% Confidence Interval (CI) for 2019 temporal validation in SSNAP and Riksstroke external validation with in-hospital mortality.

	Brier score (95% CI)		AUC (95% CI)	
	SSNAP	Riksstroke	SSNAP	Riksstroke
LR	0.078 (0.076, 0.080)	0.066 (0.065, 0.067)	0.847 (0.841, 0.853)	0.861 (0.857, 0.864)
LR with elastic net and interaction terms	0.078 (0.076, 0.080)	0.067 (0.066, 0.068)	0.850 (0.844, 0.856)	0.862 (0.859, 0.866)
XGBoost	0.077 (0.075 ,0.079)	0.066 (0.065, 0.067)	0.852 (0.846, 0.858)	0.861 (0.858, 0.865)

For peer review only

Model specification and performance

General characteristics for the development, validation and temporal validation set on all candidate variables are presented in previous study [7]. Specifications of the trained models and explanations on how to use them can be found in the repository on Github (https://github.com/WenjuanW/Risk_Prediction_of_Post-Stroke_30-day_Mortality_SSNAP_and_RiksStroke). All models performed similarly on training set and validation set indicating the models were not overfitting (Supplementary Table B) and calibrated well (Supplementary Figure A).

For the performances on 2019 temporal validation with SSNAP data (Table 2), XGBoost obtained the lowest Brier score of 0.077 (95% CI: 0.075 to 0.079) and the highest AUC of 0.852 (95% CI: 0.846 to 0.858) compared to LR with elastic net and interaction terms (Brier score: 0.078 (0.076, 0.080) and AUC: 0.850 (0.844, 0.856)) and LR (Brier score: 0.078 (0.076, 0.080) and AUC: 0.847 (0.841, 0.853)). The differences between the models were all significant (p-value < 0.001) even though small.

For the performances on external validation with Riksstroke data (Table 2) with in-hospital mortality, XGBoost achieved the lowest Brier score (0.066 (95% CI: 0.065 to 0.067) vs 0.067 (95% CI: 0.066 to 0.068) from LR with elastic net and interaction terms vs 0.066 (95% CI: 0.065 to 0.067) from LR, p-value < 0.001). In terms of AUC, XGBoost achieved the slightly better performance compared to LR with elastic net and interaction terms (0.861 (95% CI: 0.858 to 0.865) vs 0.862 (95% CI: 0.859 to 0.866), p-value = 0.008) whilst better than LR (0.861 (95% CI: 0.857 to 0.864), p-value < 0.001). The performances of the models on Riksstroke data were slightly better than on SSNAP temporal validation data. The AUC between in-hospital and all-mortality in RiksStroke were similar (Supplementary Table C and Figure B)

From the precision-recall curves (Figure 1), the models were better at predicting precision on the Riksstroke data compared to on SSNAP data. Calibration plots in Figure 1 shows good calibration for the 2019 temporal validation data in SSNAP with slightly underestimation of the risks for patients with medium risks (0.2-0.6). The models overestimated the risks for in-hospital mortality in Riksstroke for about 0.1 for medium risks patients whilst the overestimation was smaller for the all mortality predictions in Riksstroke. Decision curves on SSNAP 2019 temporal validation set and Riksstroke external validation set showed all models gained similar net benefits [20] (Supplementary Figure B).

For peer review only - http://bmjopen.bmj.com/site/about/guidelines.xhtml

For different stroke types, all models performed better on haemorrhagic patients than infarction patients on both SSNAP data (Figure 2) and Riksstroke data (Figure 3 for in-hospital mortality and Supplementary Figure C for all-mortality) in terms of AUC and area under precision-recall curves. Calibrations were similar between different stroke types (Figure 2, 3 and Supplementary Figure C).

According to the feature importance calculated from the XGBoost model (Supplementary Figure D), level of consciousness, type of stroke, age, prestroke mRS, and AF were the most important features in making the predictions which were in line with the literature. The coefficients from LR model (Supplementary Table D) also confirmed the importance of these variables.

Through the sensitivity analysis of using pre-stroke mRS=1 as the level representing 0-2 in previous stroke mRS variable in Riksstroke, we found no impact on the performances.

Figure 1: Precision-Recall curves (above) and Calibration curves (below) for all models on temporal validation of SSNAP cohort (left) and external validation using Riksstroke with in-hospital mortality (middle) and Riksstroke with all mortality (right)

Figure 2: AUC-ROC, PR-curve, and Calibration-curve for in-hospital mortalities (Haemorrhage (left) vs Ischemic (right) patients) in SSNAP temporal validation set

Figure 3: AUC-ROC, PR-curve, and Calibration-curve for in-hospital mortalities (Haemorrhage (left) vs Ischemic (right) patients) in Riksstroke

Discussion

This is the first cross-national study to develop and externally validate a ML-based risk prediction model for 30-day mortality after stroke that is suitable for use in care quality registers. Mortality is an important outcome after stroke and widely reported by stroke quality initiatives as a measure of quality and safety of acute stroke care, including in the Riksstroke

For peer review only - http://bmjopen.bmj.com/site/about/guidelines.xhtml

and SSNAP national quality registers used in this study. The results of the internal validation in the UK and the external validation in Sweden were similar and together provide evidence that the model was accurate and generalisable across countries and data sources. This is promising for developing a single model that can be used to benchmark different hospitals/organisations in different countries and make fairer comparisons of stroke outcomes across countries. But note that the UK and Sweden both have similar European patient populations, and there are many similarities between health systems. It is not as certain whether this model would be generalizable to a nation with a much more different patient population or care delivery system.

Externally validated prediction models can be valuable tools in benchmarking health care outcomes across different hospitals[1] or countries and thereby contributing to improved clinical practice. This can be achieved by comparing the standardised mortality ratio (SMR) [21] which is calculated using the observed mortality rate and the predicted risk of mortality. A higher than expected SMR may be an indicator of poorer quality care, and there is strong evidence that higher quality acute stroke care (such as stroke unit based care [22]) reduces the risk of dying after stroke. The validity of this approach is however dependent on being able to make an accurate prediction of mortality, and hence it is important that the models used to generate these predictions are as accurate as possible and have been thoroughly validated. Another use case for validated models could be identifying patients with high risk for intervention. Patients at high risk for 30-day mortality should be considered for intensive outpatient interventions, and further studied to see if this reduces their risk.

We found that the performance of the model in external validation with Riksstroke data was in fact slightly better than in the temporal validation using data from SSNAP. This may be because the models performed more accurately in patients with haemorrhagic stroke and Riksstroke patients had a higher prevalence of haemorrhage patients. Also, the model was calibrated better for all mortality than in-hospital mortality in Riksstroke. The reason for this is unclear, but one possible explanation could be that the in-hospital mortality rate in Riksstroke (10.8%) was lower than in SSNAP (12.4%) so the prediction model built from SSNAP overestimated the mortality rate in Riksstroke.

Although we could not externally validate the models with the existing stroke prognostic scores, our models showed performance accuracy similar to that of these scores in predicting in-hospital mortality. One study [23] has assessed the performance of 6 stroke prognostic

For peer review only - http://bmjopen.bmj.com/site/about/guidelines.xhtml

scores in 4237 acute ischemic stroke patients hospitalised at a Japanese stroke centre between 2012 and 2017 including existing risk score PLAN [8] with AUC (0.87 (0.85–0.90)) [23] and IScore with AUC (0.88 (0.86–0.91)) [23]. The performances in the Japanese cohort were higher compared to the original studies (PLAN: AUC 0.79, IScore: AUC 0.79–0.85) but the findings may not generalisable to other stroke populations.There have been many ML models built for predicting stroke mortality [3,24]. Unfortunately, we are unable to externally validate these as the final models were not published. The objectives for these studies were to obtain accurate prognostication of stroke for helping with therapy and rehabilitation planning. In this study, we aimed to have models that were developed in one nation and externally validated in another so the models could be used as benchmarks for evaluating quality of stroke care.

Regarding the comparisons between ML and regression based models, our study also confirmed that there is only a small performance benefit of machine learning over carefully developed logistic regression models for clinical prediction models, which was consistent with prior literature [5,7]. While the AUC between models may have been statistically significant, the differences were small and unlikely clinically significant. In this case, where a limited number of predicting features were included, LR performed similarly to ML and ML might not add much discriminatory value compared to LR. However, ML has outperformed LR in some other settings [3]. The role of machine learning in quality improvement has not been extensively explored. This study suggests that ML could have a role as part of the analysis and feedback of data about healthcare quality in specific situations without pre-specified model assumptions and restrictions in the number of included variables.

Strengths and limitations

The models were built and externally validated with very large and comprehensive stroke datasets (including data on 488,497 patients for development and validation and 128,360 patients for the external validation). Both data sources include a nearly complete population of hospitalised stroke in England and Wales (SSNAP) and Sweden (Riksstroke) reducing the risk of bias.

The models were built with robust approaches and reported according to the TRIPOD reporting guidelines with several terms adjusted for ML studies such as hyperparameter turning and final model presentation. The models were published and a have been made freely available for download for future external validation and use (GitHub repository:

For peer review only - http://bmjopen.bmj.com/site/about/guidelines.xhtml

For peer review only

https://github.com/WenjuanW/Risk_Prediction_of_Post-Stroke_30-day_Mortality_SSNAP_and_RiksStroke).

The main limitations are: 1) Death outcomes were limited to inpatient mortality, and it was not possible to ascertain deaths occurring outside hospital within 30 days for SSNAP data. Hence, the rates of 30-day mortality are likely underestimated and imprecise. But from the sensitivity analysis using all mortality from Riksstroke data, the rate of out-hospital mortality is small (2.3%) and the performance of external validation was good for both in-hospital mortality and all mortality. 2) NIHSS as a main risk predictor for mortality was not available in Riksstroke. Level of consciousness was used instead and according to previous studies Bray et al. 2014 [6], the level of consciousness component of the NIHSS provides a good approximation of the full NIHSS for mortality prediction in settings where the ability to record the full NIHSS on admission is limited. 3) Some other risk factors are not available such as imaging, congestive heart failure etc. 4) The variables were limited to the ones that are available in both SSNAP and Riksstroke. However, all variables are generally available or can be derived from available variables in stroke registries which can benefit from the models developed in this study. 5) As can be expected, there were some discrepancies in how the included variables were recorded in the two registers. Well-constructed methods were applied to handle the discrepancies and further sensitivity analysis were conducted. 6) Only XGBoost was used for developing the model from ML-based methods but as we know that generally ML methods are not outperforming statistical methods with structured data, more ML methods would not be likely to add more information due to the limited number of variables.

For peer review only

Conclusions

We have developed and externally validated a machine learning based model to predict 30 day mortality after stroke, a widely collected and reported measure of stroke quality. The development of these types of generalisable models are important in allowing for unbiased comparisons of stroke outcomes across countries, and would be well suited to support efforts to measure and compare stroke outcomes as part of quality improvement initiatives.

Declarations

For peer review only - http://bmjopen.bmj.com/site/about/guidelines.xhtml

Ethics approval and consent to participate

SSNAP has approval from the Clinical Advisory Group of the NHS Health Research Authority to collect patient-level data under section 251 of the NHS Act 2006. No additional ethical review was sought. Informed consent was not sought for the present study because Data analyses were carried out using fully anonymised datasets from SSNAP.

The external validation using Riksstroke was approved by the Swedish Ethical Review Authority (reference no. 2021-06152-01). In accordance with the Personal Data Act (Swedish law No. SFS 1998:204), no informed consent is needed to collect data from medical charts and inpatient records for quality registers.

Data sharing statement

The data that support the findings of this study are available from SSNAP (www.strokeaudit.org) and Riksstroke (www.riksstroke.org) but restrictions apply to the availability of these data, which were used under license for the current study, and so are not publicly available. Data are however available from the SSNAP and Riksstroke upon reasonable request and with permission of HQIP and Riksstroke, respectively.

Competing interests

The authors declare that they have no competing interests.

Funding

CDW, VC, and WW acknowledge the financial support from the Health Foundation. CDW acknowledge support from the National Institute for Health Research (NIHR) Biomedical Research Centre (BRC) based at Guy's and St Thomas' National Health Service (NHS) Foundation Trust and King's College London, and the NIHR Collaboration for Leadership in Applied Health Research and Care (ARC) South London at King's College Hospital NHS Foundation Trust. VC is supported by the National Institute for Health Research (NIHR)

For peer review only - http://bmjopen.bmj.com/site/about/guidelines.xhtml

Biomedical Research Centre based at Guy's and St Thomas' National Health Service Foundation Trust and King's College London, and the Public Health and Multi-morbidity Theme of the National Institute for Health Research's Applied Research Collaboration (ARC) South London. VC is also supported by the EPSRC CONSULT grant (EP/P010105/1). The views expressed are those of the authors and not necessarily those of the NHS, the BRC or ARC.

Authors' contributions

Study design: WW, ME, BB

Literature review: WW, JO, ME, BB

Figures: WW, JO

Data cleaning and preprocessing: WW, JO

Model developing and internal validation (UK): WW, BB

External validation (Sweden): JO, ME

Data interpretation: WW, JO, ME, BB, CW, VC

Writing - original draft: WW, JO

Writing - review & editing: WW, BB, ME, JO, CW, VC

Supervision: BB (UK), ME (Sweden)

Funding acquisition (UK): BB, CW, VC

Project administration (UK): WW, BB, VC

References

1. Jarman B, Gault S, Alves B, et al. Explaining differences in English hospital death rates using routinely collected data. *BMJ*. 1999;318(7197):1515-1520. doi:10.1136/bmj.318.7197.1515
2. Cadilhac DA, Kim J, Lannin NA, et al. National stroke registries for monitoring and improving the quality of hospital care: A systematic review. *Int J Stroke Off J Int Stroke Soc*. 2016;11(1):28-40. doi:10.1177/1747493015607523

For peer review only - http://bmjopen.bmj.com/site/about/guidelines.xhtml

3. Wang W, Kiik M, Peek N, et al. A systematic review of machine learning models for predicting outcomes of stroke with structured data. *PLOS ONE*. 2020;15(6):e0234722. doi:10.1371/journal.pone.0234722
4. Shah P, Kendall F, Khozin S, et al. Artificial intelligence and machine learning in clinical development: a translational perspective. *Npj Digit Med*. 2019;2(1):1-5. doi:10.1038/s41746-019-0148-3
5. Christodoulou E, Ma J, Collins GS, Steyerberg EW, Verbakel JY, Van Calster B. A systematic review shows no performance benefit of machine learning over logistic regression for clinical prediction models. *J Clin Epidemiol*. 2019;110:12-22. doi:10.1016/j.jclinepi.2019.02.004
6. Bray BD, Campbell J, Cloud GC, et al. Derivation and external validation of a case mix model for the standardized reporting of 30-day stroke mortality rates. *Stroke*. 2014;45(11):3374-3380. doi:10.1161/STROKEAHA.114.006451
7. Wang W, Rudd AG, Wang Y, et al. Risk prediction of 30-day mortality after stroke using machine learning: a nationwide registry-based cohort study. *BMC Neurol*. 2022;22(1):195. doi:10.1186/s12883-022-02722-1
8. O'Donnell MJ, Fang J, D'Uva C, et al. The PLAN score: a bedside prediction rule for death and severe disability following acute ischemic stroke. *Arch Intern Med*. 2012;172(20):1548-1556. doi:10.1001/2013.jamainternmed.30
9. Saposnik G, Kapral MK, Liu Y, et al. IScore: a risk score to predict death early after hospitalization for an acute ischemic stroke. *Circulation*. 2011;123(7):739-749. doi:10.1161/CIRCULATIONAHA.110.983353
10. Collins GS, Reitsma JB, Altman DG, Moons KG. Transparent reporting of a multivariable prediction model for individual prognosis or diagnosis (TRIPOD): the TRIPOD Statement. *BMC Med*. 2015;13(1):1. doi:10.1186/s12916-014-0241-z
11. Asplund K, Hulter Åsberg K, Appelros P, et al. The Riks-Stroke story: building a sustainable national register for quality assessment of stroke care. *Int J Stroke Off J Int Stroke Soc*. 2011;6(2):99-108. doi:10.1111/j.1747-4949.2010.00557.x
12. Fonarow GC, Saver JL, Smith EE, et al. Relationship of national institutes of health stroke scale to 30-day mortality in medicare beneficiaries with acute ischemic stroke. *J Am Heart Assoc*. 2012;1(1):42-50. doi:10.1161/JAHA.111.000034
13. Eriksson M, Appelros P, Norrving B, Terént A, Stegmayr B. Assessment of functional outcome in a national quality register for acute stroke: can simple self-reported items be transformed into the modified Rankin Scale? *Stroke*. 2007;38(4):1384-1386. doi:10.1161/01.STR.0000260102.97954.9c
14. Azur MJ, Stuart EA, Frangakis C, Leaf PJ. Multiple imputation by chained equations: what is it and how does it work? *Int J Methods Psychiatr Res*. 2011;20(1):40-49. doi:10.1002/mpr.329
15. Baneshi MR, Talei AR. Does the Missing Data Imputation Method Affect the Composition and Performance of Prognostic Models? *Iran Red Crescent Med J*. 2012;14(1):31.
16. Zou H, Hastie T. Regularization and Variable Selection via the Elastic Net. *J R Stat Soc Ser B Stat Methodol*. 2005;67(2):301-320.
17. Chen T, Guestrin C. XGBoost: A Scalable Tree Boosting System. In: *Proceedings of the 22nd ACM SIGKDD International Conference on Knowledge Discovery and Data Mining*. KDD '16. Association for Computing Machinery; 2016:785-794. doi:10.1145/2939672.2939785
18. Gerds TA, Cai T, Schumacher M. The performance of risk prediction models. *Biom J Biom Z*. 2008;50(4):457-479. doi:10.1002/bimj.200810443
19. Van Calster B, McLernon DJ, van Smeden M, et al. Calibration: the Achilles heel of predictive analytics. *BMC Med*. 2019;17(1):230. doi:10.1186/s12916-019-1466-7
20. Vickers AJ, Elkin EB. Decision curve analysis: a novel method for evaluating prediction models. *Med Decis Mak Int J Soc Med Decis Mak*. 2006;26(6):565-574. doi:10.1177/0272989X06295361
21. Roessler M, Schmitt J, Schoffer O. Can we trust the standardized mortality ratio? A formal analysis and evaluation based on axiomatic requirements. *PLoS ONE*. 2021;16(9):e0257003. doi:10.1371/journal.pone.0257003
22. Organised inpatient (stroke unit) care. doi:10.1002/14651858.CD000197.pub4

For peer review only - http://bmjopen.bmj.com/site/about/guidelines.xhtml

23. Matsumoto K, Nohara Y, Soejima H, Yonehara T, Nakashima N, Kamouchi M. Stroke Prognostic Scores and Data-Driven Prediction of Clinical Outcomes After Acute Ischemic Stroke. *Stroke*. 2020;51(5):1477-1483. doi:10.1161/STROKEAHA.119.027300
24. Schwartz L, Anteby R, Klang E, Soffer S. Stroke mortality prediction using machine learning: systematic review. *J Neurol Sci*. 2023;444:120529. doi:10.1016/j.jns.2022.120529

For peer review only

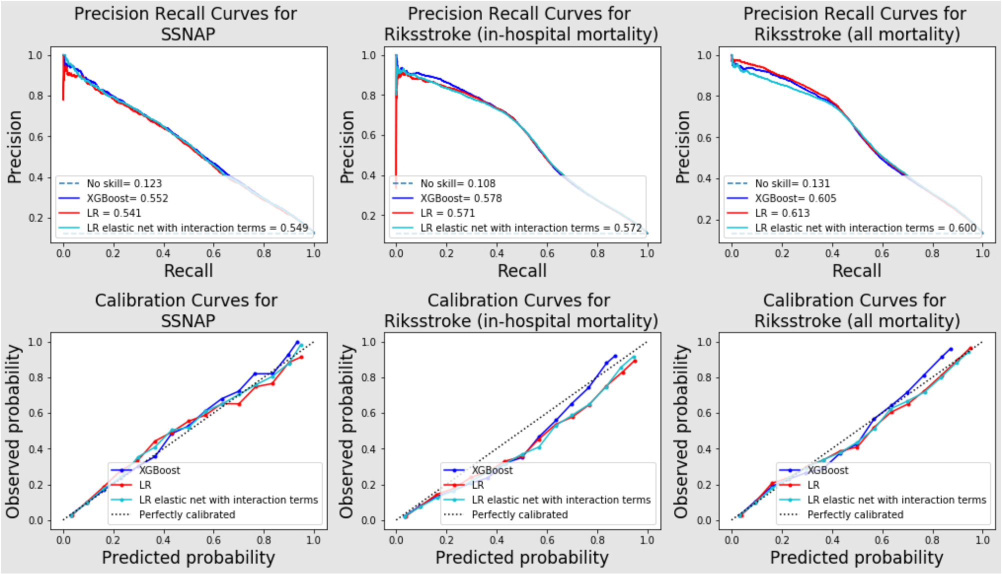

Figure 1: Precision-Recall curves (above) and Calibration curves (below) for all models on temporal validation of SSNAP cohort (left) and external validation using Riksstroke with in-hospital mortality (middle) and Riksstroke with all mortality (right)

357x204mm (72 x 72 DPI)

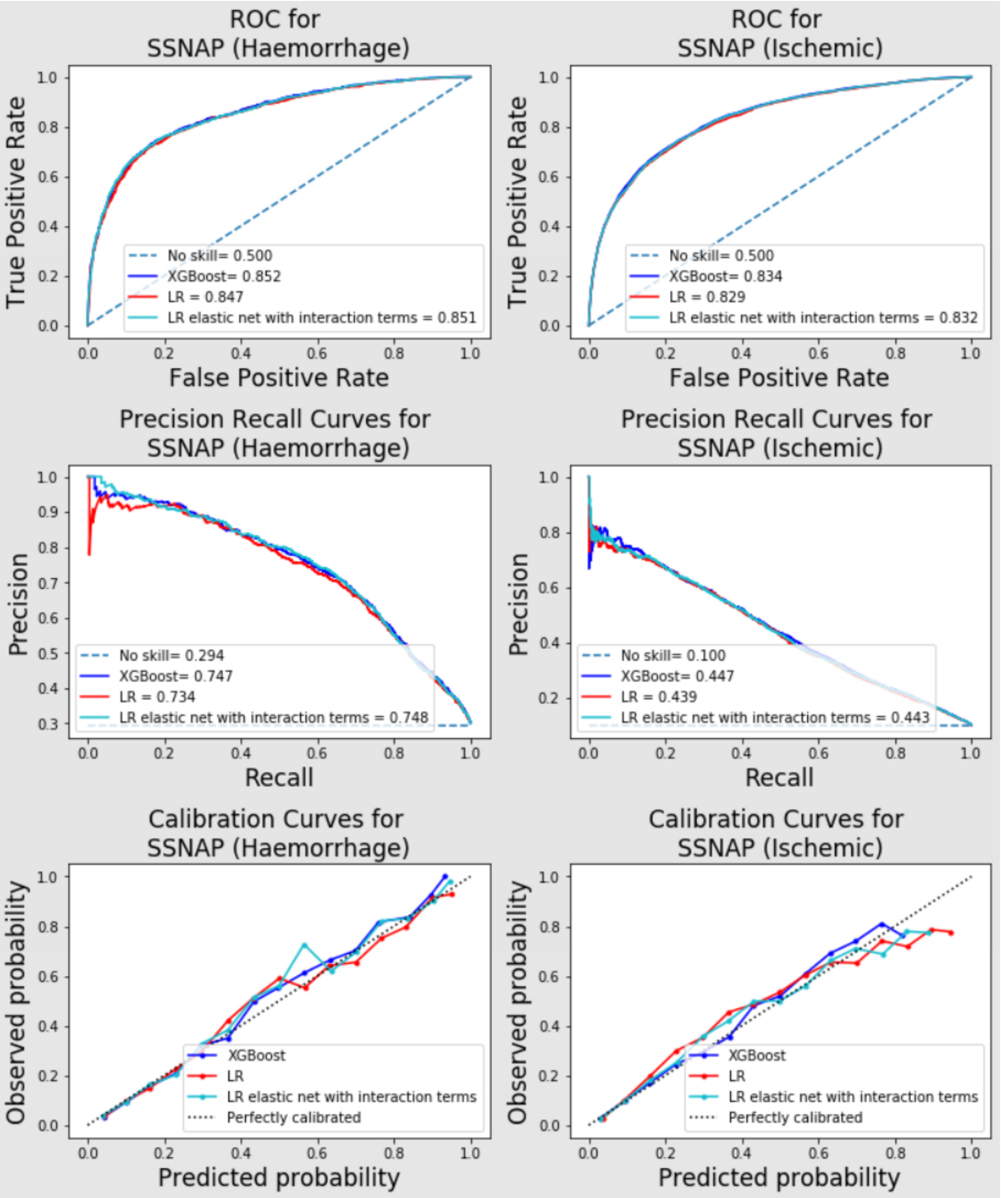

Figure 2: AUC-ROC, PR-curve, and Calibration-curve for in-hospital mortalities (Haemorrhage (left) vs Ischemic (right) patients) in SSNAP temporal validation set

319x382mm (72 x 72 DPI)

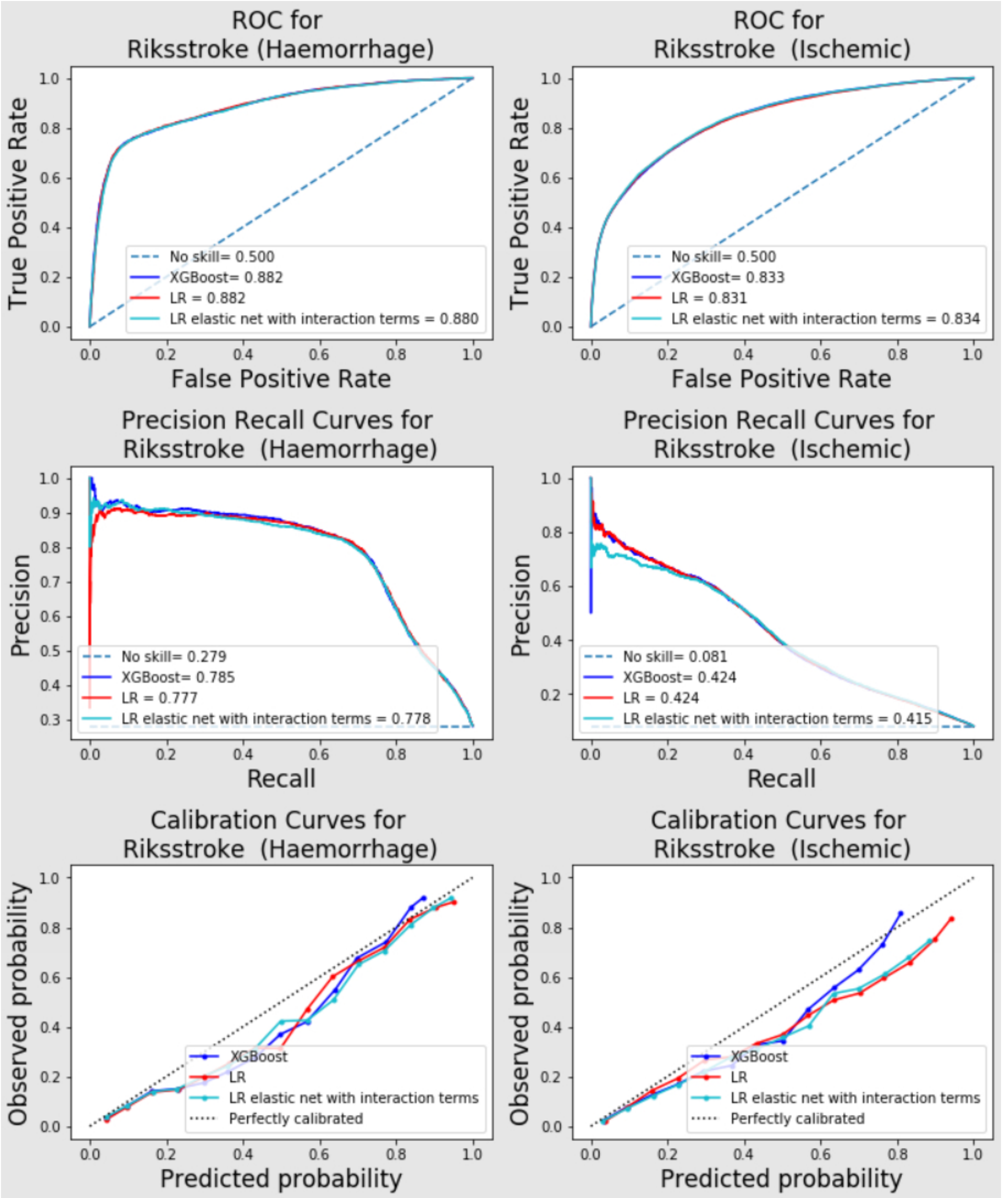

Figure 3: AUC-ROC, PR-curve, and Calibration-curve for in-hospital mortalities (Haemorrhage (left) vs Ischemic (right) patients) in Riksstroke

319x382mm (72 x 72 DPI)

Supplementary Material

Table A, Missing values in the variables and the imputation methods

	SSNAP		Riksstroke (in-hospital mortality)	
	**Missing (%)**	**Imputation method**	**Missing (%)**	**Imputation method**
Age groups	-	-	-	-
Male	-	-	-	-
Hypertension	-	-	0.5%	Multiple imputation
AF	-	-	0.2%	Multiple imputation
Diabetes	-	-	0.3%	Multiple imputation
Previous Stroke or TIA	-	-	0.3%	Multiple imputation
NIHSS at arrival	-	-	42.9%	Not used in the model
Level of consciousness	-	-	1.5%	Multiple imputation after splitting the non alert level to two levels
Pre-stroke mRS			5.0%	multiple imputation

For peer review only

anticoagulation prior to stroke if AF	58.9%	Unknown as a new category	-	-
Type of stroke	0.7%	Unknown as a new category	-	-

Abbreviation: AF: Atrial Fibrillation; TIA: transient ischaemic attack; NIHSS: National Institutes of Health Stroke Scale; mRS: modified Rankin Scale.

Table B: Brier score, AUC with 95% Confidence Interval (CI) for SSNAP development set and internal validation set.

	Brier score (95% CI)		AUC (95% CI)	
	SSNAP development set	SSNAP validation set	SSNAP development set	SSNAP validation set
LR	0.081 (0.080, 0.082)	0.079 (0.78, 0.81)	0.843 (0.841, 0.845)	0.845 (0.841, 0.849)
LR with elastic net and interaction terms	0.080 (0.079, 0.081)	0.078 (0.77, 0.80)	0.845 (0.843, 0.847)	0.848 (0.844, 0.851)
XGBoost	0.079 (0.079, 0.080)	0.078 (0.77, 0.79)	0.848 (0.846, 0.850)	0.849 (0.845, 0.853)

For peer review only

Table C, Brier score, AUC with 95% Confidence Interval (CI) for 2019 temporal validation and RiksStroke external validation with in-hospital mortality and all mortality.

	Brier score (95% CI)			AUC (95% CI)		
	SSNAP	RiksStroke (in-hospital mortality)	RiksStroke (all mortality)	SSNAP	RiksStroke (in-hospital mortality)	RiksStroke (all mortality)
LR	0.078 (0.076, 0.080)	0.066 (0.065, 0.067)	0.076 (0.075, 0.077)	0.847 (0.841, 0.853)	0.861 (0.857, 0.864)	0.859 (0.856, 0.862)
LR with elastic net and interaction terms	0.078 (0.076, 0.080)	0.067 (0.066, 0.068)	0.077 (0.076, 0.078)	0.850 (0.844, 0.856)	0.862 (0.859, 0.866)	0.861 (0.858, 0.864)
XGBoost	0.077 (0.075 ,0.079)	0.066 (0.065, 0.067)	0.077 (0.076, 0.078)	0.852 (0.846, 0.858)	0.861 (0.858, 0.865)	0.860 (0.857, 0.863)

Table D, the coefficients of variables from logistic regression

Variables	Coefficients
**loss of consciousness**	**1.26**

For peer review only

type of stroke Haemorrhage	0.92
Inpatient at time of stroke	0.47
atrial fibrillation	0.46
type of stroke Unknown	0.32
Age (Groups by 5)	0.22
hour of admission 04 to 08	0.17
previous stroke/TIA	-0.16
prestroke mRS	0.16
hour of admission 12 to 16	-0.14
hypertension	-0.12
Male	0.11
admission Friday	-0.10
diabetes	0.10
hour of admission 16 to 20	-0.09
admission Monday	-0.08
prior anticoagulation if AF Nobut	0.07
admission Tuesday	-0.05
admission Wednesday	-0.05

For peer review only

**admission Thursday**	**-0.05**
**admission Saturday**	**-0.03**
**hour of admission 08 to 12**	**-0.03**
**prior anticoagulation if AF Yes**	**-0.02**
**hour of admission 20 to 24**	**0.02**
**prior anticoagulation if AF Unknown**	**-0.005**

Abbreviation: AF: Atrial Fibrillation; TIA: transient ischaemic attack; mRS: modified Rankin Scale.

For peer review only

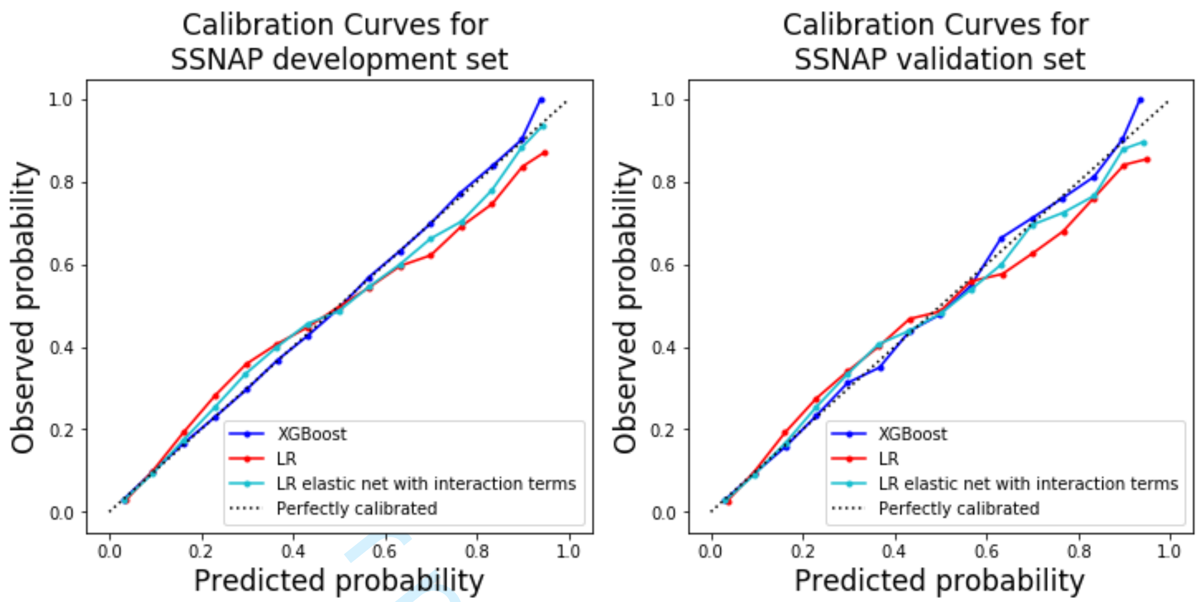

Figure A: Calibration curves for all models on development set and validation set of SSNAP cohort.

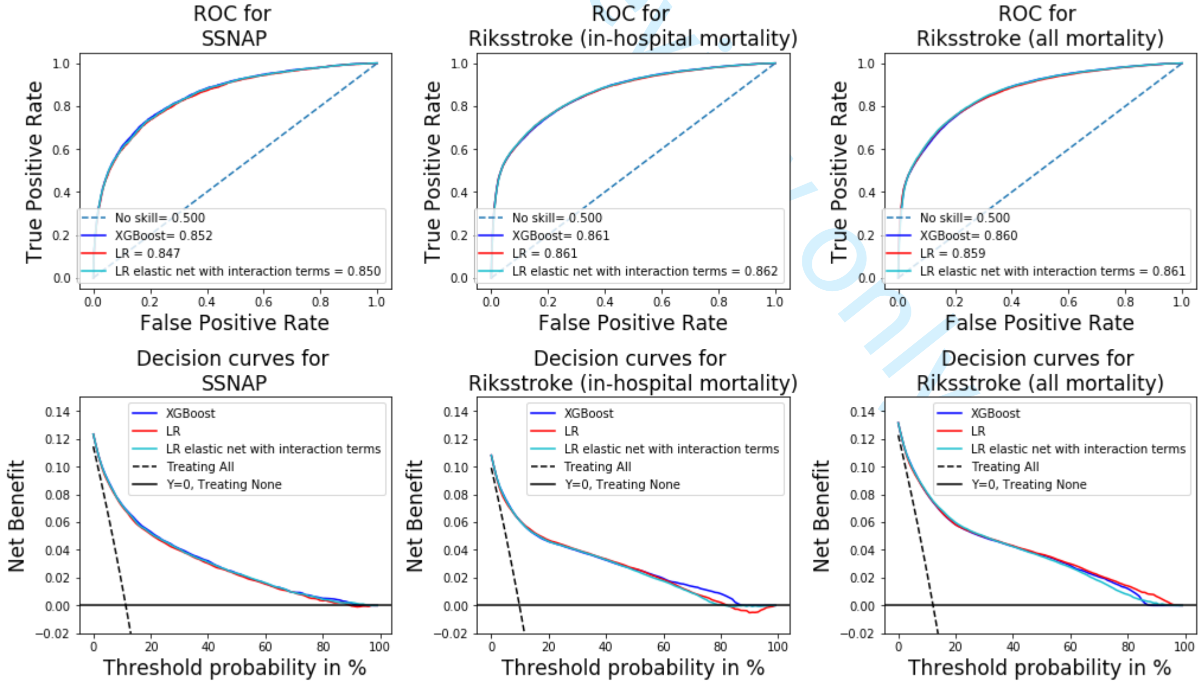

Figure B: ROC and decision curves for all models on 2019 temporal validation of SSNAP cohort (left) and external validation using Riksstroke with in-hospital mortality (middle) and all mortality (right)

For peer review only - http://bmjopen.bmj.com/site/about/guidelines.xhtml

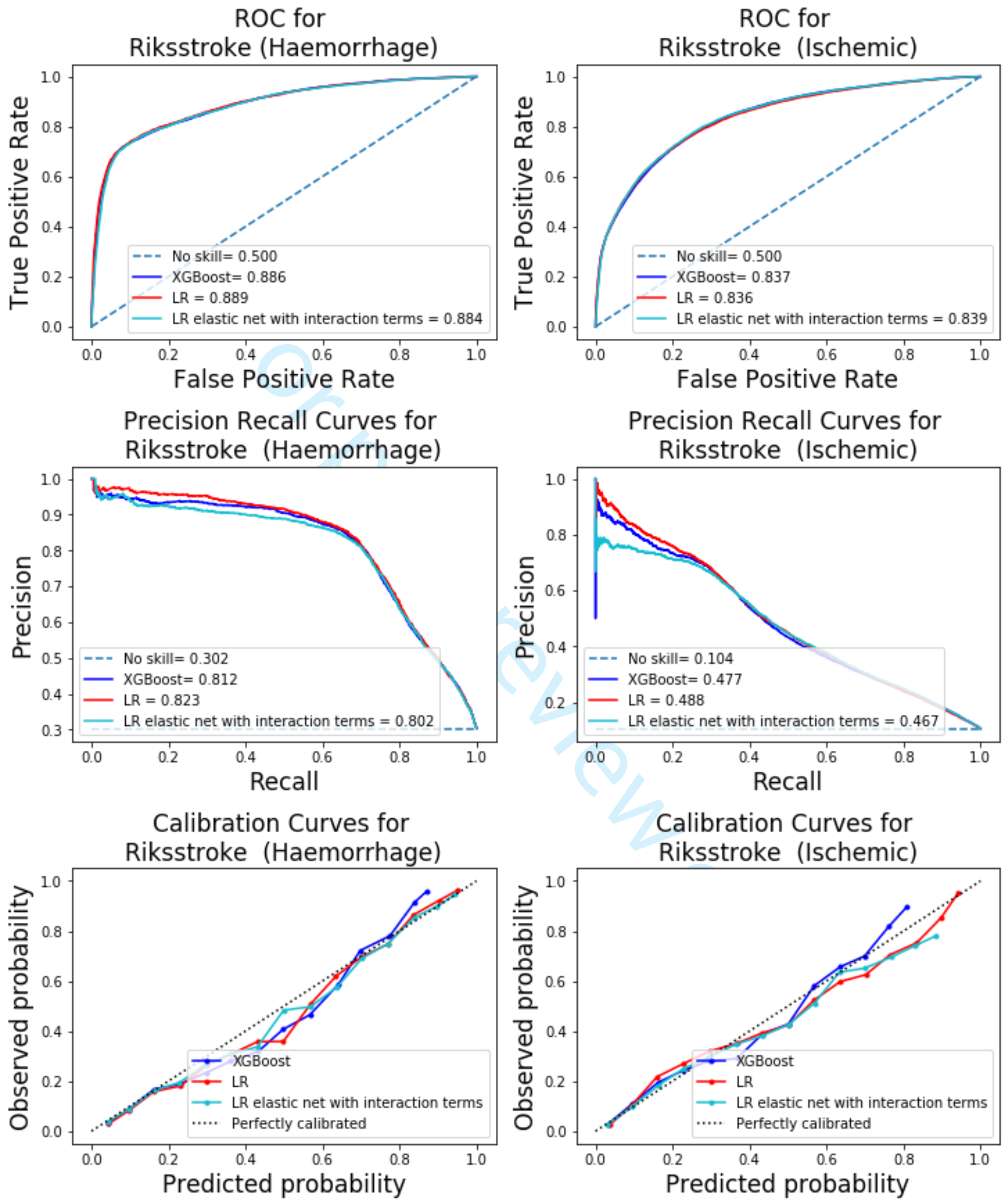

Figure C: AUC-ROC, PR-curve, and Calibration-curve for all mortalities (Haemorrhage (left) vs Ischemic (right) patients) in Riksstroke

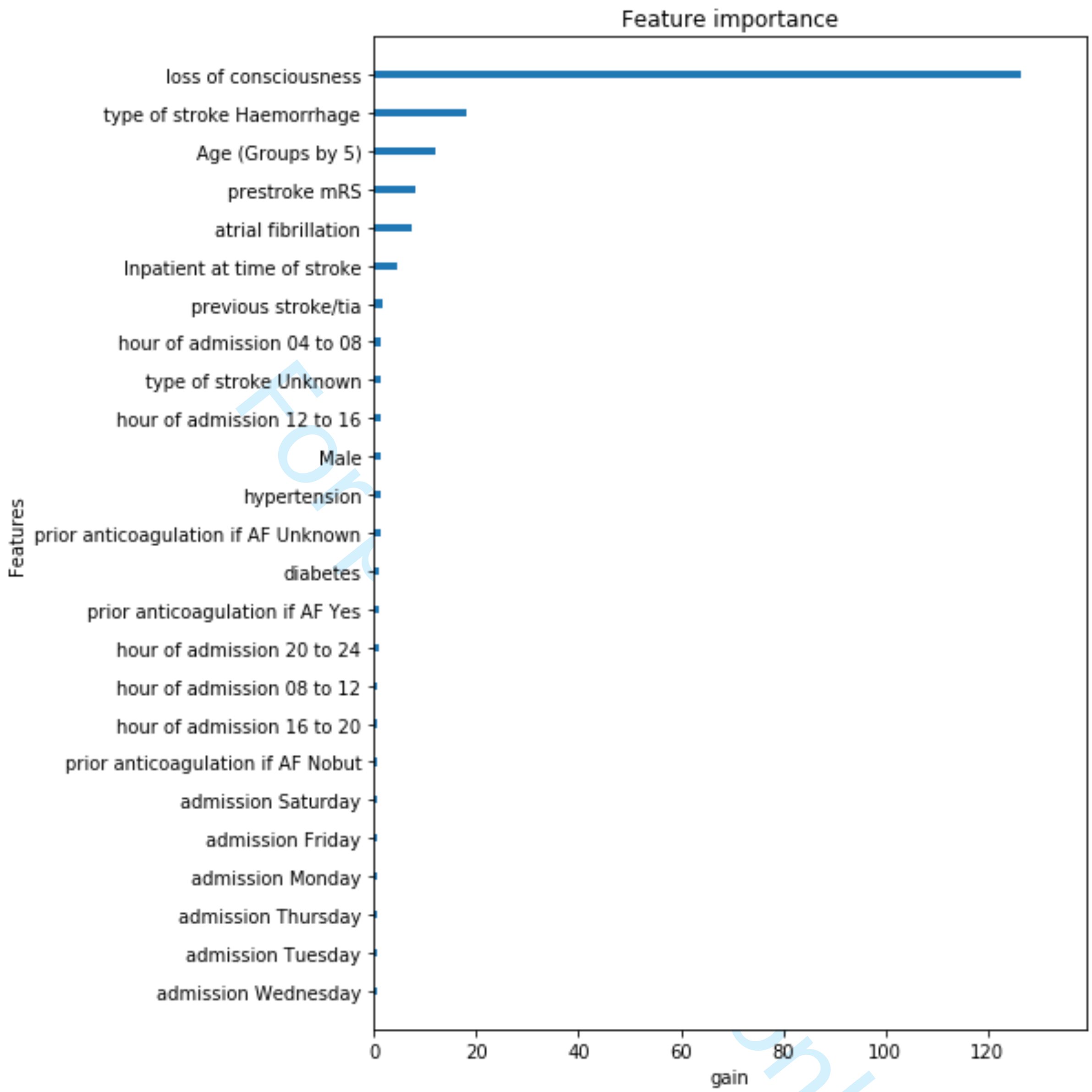

Figure D: Feature importance from XGBoost using gain as measurement of feature importance

Table: Transparent reporting of a multivariable prediction model for individual prognosis or diagnosis (TRIPOD) checklist

Section/ Topic	m	Checklist Item	Page
**Title and abstract**			
Title	1	Identify the study as developing and/or validating a multivariable prediction model, the target population, and the outcome to be predicted.	0
Abstract	2	Provide a summary of objectives, study design, setting, participants, sample size, predictors, outcome, statistical analysis, results, and conclusions.	1
**Introduction**			
Background and objectives	3a	Explain the medical context (including whether diagnostic or prognostic) and rationale for developing or validating the multivariable prediction model, including references to existing models.	4
	3b	Specify the objectives, including whether the study describes the development or validation of the model or both.	4-5
**Methods**			
Source of data	4a	Describe the study design or source of data (e.g., randomized trial, cohort, or registry data), separately for the development and validation data sets, if applicable.	6

For peer review only - http://bmjopen.bmj.com/site/about/guidelines.xhtml

	4b	Specify the key study dates, including start of accrual; end of accrual; and, if applicable, end of follow-up.	6
Participants	5a	Specify key elements of the study setting (e.g., primary care, secondary care, general population) including number and location of centres.	6
	5b	Describe eligibility criteria for participants.	6
	5c	Give details of treatments received, if relevant.	-
Outcome	6a	Clearly define the outcome that is predicted by the prediction model, including how and when assessed.	7
	6b	Report any actions to blind assessment of the outcome to be predicted.	-
Predictors	7a	Clearly define all predictors used in developing or validating the model, including how and when they were measured.	7-8
	7b	Report any actions to blind assessment of predictors for the outcome and other predictors.	-
Sample size	8	Explain how the study size was arrived at.	6
Missing data	9	Describe how missing data were handled (e.g., complete-case analysis, single imputation, multiple imputation) with details of any imputation method.	8
Statistical analysi	10a	Describe how predictors were handled in the analyses.	7-8
	10b	Specify type of model, all model-building procedures and method for internal validation.	8-9

For peer review only

s method s	10d	Specify all measures used to assess model performance and, if relevant, to compare multiple models.	9-10
Risk groups	11	Provide details on how risk groups were created, if done.	10
**Results**			
Particip ants	13a	Describe the flow of participants through the study, including the number of participants with and without the outcome and, if applicable, a summary of the follow-up time. A diagram may be helpful.	-
	13b	Describe the characteristics of the participants (basic demographics, clinical features, available predictors), including the number of participants with missing data for predictors and outcome.	11-13
Model develo pment	14a	Specify the number of participants and outcome events in each analysis.	11
	14b	If done, report the unadjusted association between each candidate predictor and outcome.	-
Model specific ation	15a	Present the full prediction model to allow predictions for individuals.	14
	15b	Explain how to the use the prediction model.	14
Model perfor mance	16	Report performance measures (with CIs) for the prediction model.	14-15
**Discussion**			

For peer review only - http://bmjopen.bmj.com/site/about/guidelines.xhtml

Limitations	18	Discuss any limitations of the study (such as nonrepresentative sample, few events per predictor, missing data).	17-18
Interpretation	19b	Give an overall interpretation of the results, considering objectives, limitations, and results from similar studies, and other relevant evidence.	15-18
Implications	20	Discuss the potential clinical use of the model and implications for future research.	15-17
**Other information**			
Supplementary information	21	Provide information about the availability of supplementary resources, such as study protocol, Web calculator, and data sets.	Yes
Funding	22	Give the source of funding and the role of the funders for the present study.	Yes

For peer review only - http://bmjopen.bmj.com/site/about/guidelines.xhtml
